# Genetic Loci Associated With COVID-19 Positivity and Hospitalization in White, Black, and Hispanic Veterans of the VA Million Veteran Program

**DOI:** 10.3389/fgene.2021.777076

**Published:** 2022-02-03

**Authors:** Gina M. Peloso, Catherine Tcheandjieu, John E. McGeary, Daniel C. Posner, Yuk-Lam Ho, Jin J. Zhou, Austin T. Hilliard, Jacob Joseph, Christopher J. O’Donnell, Jimmy T. Efird, Dana C. Crawford, Wen-Chih Wu, Mehrdad Arjomandi, VA Million Veteran Program COVID-19 Science Initiative, Yan V. Sun, Themistocles L Assimes, Jennifer E. Huffman

**Affiliations:** ^1^ Massachusetts Veterans Epidemiology Research and Information Center, VA Boston Healthcare System, Boston, MA, United States; ^2^ Department of Biostatistics, Boston University School of Public Health, Boston, MA, United States; ^3^ VA Palo Alto Healthcare System, Palo Alto, CA, United States; ^4^ Division of Cardiovascular Medicine, Department of Medicine, Stanford University School of Medicine, Stanford, CA, United States; ^5^ Stanford Cardiovascular Institute, Stanford University, Stanford, CA, United States; ^6^ Providence VA Healthcare System, Providence, RI, United States; ^7^ Department of Psychiatry and Human Behavior, Brown University, Providence, RI, United States; ^8^ Phoenix VA Health Care System, Phoenix, AZ, United States; ^9^ Department of Medicine, David Geffen School of Medicine, University of California, Los Angeles, CA, United States; ^10^ Cardiology Section, VA Boston Healthcare System, Boston, MA, United States; ^11^ Department of Medicine, Brigham and Women’s Hospital, Boston, MA, United States; ^12^ Cooperative Studies Program Epidemiology Center-Durham, Durham VA Health Care System, Durham, NC, United States; ^13^ Louis Stokes Cleveland VA Medical Center, Cleveland, OH, United States; ^14^ Department of Population and Quantitative Health Sciences, Case Western Reserve University, Cleveland, OH, United States; ^15^ Department of Genetics and Genome Sciences, Case Western Reserve University, Cleveland, OH, United States; ^16^ Cleveland Institute for Computational Biology, Case Western Reserve University, Cleveland, OH, United States; ^17^ Department of Medicine, Alpert Medical School, Brown University, Providence, RI, United States; ^18^ Medical Service, San Francisco VA Medical Center, San Francisco, CA, United States; ^19^ Department of Medicine, University of California, San Francisco, San Francisco, CA, United States; ^20^ Atlanta VA Health Care System, Decatur, GA, United States; ^21^ Department of Epidemiology, Emory University Rollins School of Public Health, Atlanta, GA, United States

**Keywords:** COVID-19, genome-wide association study, million veteran program, hospitalization, ABO

## Abstract

SARS-CoV-2 has caused symptomatic COVID-19 and widespread death across the globe. We sought to determine genetic variants contributing to COVID-19 susceptibility and hospitalization in a large biobank linked to a national United States health system. We identified 19,168 (3.7%) lab-confirmed COVID-19 cases among Million Veteran Program participants between March 1, 2020, and February 2, 2021, including 11,778 Whites, 4,893 Blacks, and 2,497 Hispanics. A multi-population genome-wide association study (GWAS) for COVID-19 outcomes identified four independent genetic variants (rs8176719, rs73062389, rs60870724, and rs73910904) contributing to COVID-19 positivity, including one novel locus found exclusively among Hispanics. We replicated eight of nine previously reported genetic associations at an alpha of 0.05 in at least one population-specific or the multi-population meta-analysis for one of the four MVP COVID-19 outcomes. We used rs8176719 and three additional variants to accurately infer ABO blood types. We found that A, AB, and B blood types were associated with testing positive for COVID-19 compared with O blood type with the highest risk for the A blood group. We did not observe any genome-wide significant associations for COVID-19 severity outcomes among those testing positive. Our study replicates prior GWAS findings associated with testing positive for COVID-19 among mostly White samples and extends findings at three loci to Black and Hispanic individuals. We also report a new locus among Hispanics requiring further investigation. These findings may aid in the identification of novel therapeutic agents to decrease the morbidity and mortality of COVID-19 across all major ancestral populations.

## Introduction

Host-pathogen interactions are complex, dynamic processes determined by individual and interactive host and pathogen genomic and environmental factors. Differences in either the host or pathogen can result in immediate variability of disease susceptibility or expression, as evidenced by epidemics such as HIV/AIDS (Dean et al., 1996; Samson et al., 1996). For emerging infectious diseases such as coronavirus disease 2019 (COVID-19) resulting from severe acute respiratory syndrome coronavirus 2 (SARS-CoV-2) infection, the timely understanding of these complex genomic interactions has the potential to identify strategies for and predict responses to host treatments and to reveal plausible alternative strategies in anticipation of inevitable pathogen evolution (Khor and Hibberd, 2012; Kwok et al., 2021).

SARS-CoV-2 has caused symptomatic COVID-19 and widespread death across the globe since its initial identification in December 2019 in Wuhan, China. While a large fraction of the population has been infected with the virus, many individuals were asymptomatic or minimally symptomatic (Arons et al., 2020; Byambasuren et al., 2020; Sutton et al., 2020; Oran and Topol, 2021). Of those with symptomatic COVID-19, marked variability in disease presentation, morbidity, and mortality is apparent, but the underlying factors that explain or predict disease course are not completely understood. Risk factors for the development of symptomatic and severe disease include older age, male sex, obesity, and comorbidities, such as chronic kidney disease and heart failure (Goyal et al., 2020; Grasselli et al., 2020; Gu et al., 2020; Ioannou et al., 2020; Petrilli et al., 2020; Wu et al., 2020; Zhou et al., 2020). Race and ethnicity, which are variables of social construct without biological meaning, have also been implicated in susceptibility to COVID-19, with substantially higher rates of cases, hospitalization, and death reported among persons of Black or African American race and/or Hispanic ethnicity (Garg et al., 2020; Sze et al., 2020; Wu et al., 2020). However, race and ethnicity are also markers for other underlying conditions that affect health, including socioeconomic status, access to and utilization of health care, and occupational exposure to the virus. Consequently, their association may not be due to or reflect biological susceptibility to infection or COVID-19 illness (Vahidy et al., 2020; Webb Hooper et al., 2020).

The infectiousness of SARS-CoV-2 driven by an extensive and unpredictable incubation period combined with a wide range of resulting disease severity has made COVID-19 quickly amenable to genomic studies given the large number of unvaccinated, infected individuals in a short period of time. In this report, we describe the findings from a genome-wide association study (GWAS) of the United States Department of Veterans Affairs (VA) Million Veteran Program (MVP) (Gaziano et al., 2016). The aim was to examine the association of host genetic variations with 1) COVID-19 positivity (documented by a PCR test result for SARS-CoV-2 infection) and 2) susceptibility to develop a severe disease (defined as hospitalization, ICU admission, and or death). MVP represents the single largest genetically informative dataset with a substantial representation of US minority populations, linked to data on SARS-CoV-2 testing and clinical outcomes. Examining SARS-CoV-2-related phenotypes in MVP may provide important insights into population-specific and multi-population genetic architecture that possibly contribute to the biological basis for the differential susceptibility observed in Black and Hispanic Americans, in contrast to large consortia that rely on data collected in Whites only. The diversity of the MVP and difference in linkage disequilibrium patterns across genetic ancestral groups may also provide better resolution of regions associated with SARS-CoV-2 needed to identify genes or biological systems contributing to positivity, morbidity, and mortality from this virus.

## Material and Methods

### Study Participants

The VA Million Veteran Program (MVP) is an ongoing longitudinal study that began in 2011 to study genetic and non-genetic determinants of health and disease among United States Veterans (Gaziano et al., 2016). We included MVP participants who had genetic data and EHR-extracted COVID-19 related phenotype data available and who were alive as of February 29, 2020.

Demographic and clinical characteristics were obtained from the VA EHR housed within the VA’s Corporate Data Warehouse (CDW) and the MVP central data repository, curated EHR, and survey data available only for MVP research studies. Age and sex for participants were obtained from the MVP Baseline Survey and supplemented with patient health records from CDW when self-reported demographics were not available.

MVP received ethical/study protocol approval from the VA Central Institutional Review Board, and informed consent was obtained for all participants.

### Genetic Data, Quality Control, and Imputation

Study participants were genotyped using a customized Affymetrix Axiom Biobank Array (Klarin et al., 2018; Hunter-Zinck et al., 2020), and imputation was performed to a hybrid imputation panel comprised of the African Genome Resources panel (https://imputation.sanger.ac.uk/?about=1#referencepanels) and 1000 Genomes (p3v5).

Population-specific principal components (PCs) were computed using EIGENSOFT v.6 (Price et al., 2006). The harmonized race/ethnicity and genetic ancestry (HARE) approach was used to assign individuals to three mutually exclusive groups: 1) non-Hispanic White (White), 2) non-Hispanic Black (Black), and 3) Hispanic or Latino (Hispanic) (Fang et al., 2019). Kinship was inferred using KING v.2.0 (Manichaikul et al., 2010). For each pair of relatives (kinship coefficient ≥0.0884), one individual was excluded, preferentially retaining those who tested positive for SARS-CoV-2.

### COVID-19 Definitions

Cases of COVID-19 among MVP participants were identified using an algorithm developed by the VA, the COVID National Surveillance Tool (NST) (Chapman et al., 2020). COVID-19-related hospitalizations were defined as admissions from 7 days before up to 30 days after a patient’s first positive test for SARS-CoV-2. Among the genotyped 631,019 MVP participants assigned to one of the three mutually exclusive HARE groups, we excluded participants who died before March 1, 2020 (*n* = 96,807), and one participant from each pair of related individuals (*n* = 21,176). Among the remaining 513,036 participants, 19,168 tested positive for SARS-CoV-2 between March 1, 2020, and February 2, 2021, a timeframe that represents the first year of the pandemic in the US prior to widespread access to SARS-CoV-2 vaccines and the Delta variant sweep.

### ABO Blood Type

ABO blood type calling was inferred using four genetic variants: rs8176746, rs507666, rs687289, and rs8176719 (Pare et al., 2008; Severe Covid-19 GWAS Group et al., 2020). All variants were imputed with good quality (imputation r^2^ > 0.99 overall and in each HARE-assigned group apart from rs8176719, which had r^2^ > 0.95). To evaluate the accuracy of ABO blood type inferred by genotype, we calculated the concordance between genotype inferred ABO blood type and the serology-based determination for the subset of 532 genotyped MVP participants who underwent ABO typing using serologic tests during clinical care.

### Statistical Analysis

We performed single variant association between imputed variants and four outcomes: 1) COVID-19 positivity as defined by positive COVID-19 test compared with all other MVP participants (POS *vs*. POP); 2) individuals who were hospitalized for COVID-19 compared with all other MVP participants, including individuals who tested positive for COVID-19 but were not hospitalized (HOS *vs*. POP); 3) individuals who were hospitalized for COVID-19 compared with individuals who tested positive for COVID-19 but were not hospitalized (HOS *vs*. NOT); and 4) individuals who were hospitalized for COVID-19 with high-flow oxygen or died of COVID-19 (severe COVID-19) compared with all other MVP participants (SEV *vs*. POP). Participants with missing data were excluded from analyses.

Logistic regression was applied in PLINK v2 (Chang et al., 2015), adjusting for age, age^2^, sex, age*sex, and 15 population-specific PCs analysed within each of the HARE-assigned groups. Variants with population-specific minor allele frequency <0.5% or imputation quality (r^2^) < 0.3 were excluded prior to analysis. Fixed-effects meta-analysis was performed across HARE-assigned groups using GWAMA (Magi and Morris, 2010). Genome-wide significance was determined using the common threshold (p < 5 × 10^−08^) in both the multi-population meta-analysis and the population-specific analyses.

COVID-19 Host Genetics Initiative (HGI) summary statistics (release 5, excluding MVP, and 23 and Me) were used for replication. We applied Multi-marker Analysis of GenoMic Annotation (MAGMA) v1.09 for gene-based analysis, as implemented in FUMA (de Leeuw et al., 2015; Watanabe et al., 2017; de Leeuw et al., 2018) using the 1000 Genomes Phase 3 European reference panel and a window size of 10 kb ± the gene start and end. MAGMA gene-set analyses were run on 10,678 gene sets (curated gene sets: 4,761; GO terms: 5,917) from MsigDB v6.2.

The association between ABO blood type and each of the four COVID-19 outcomes was tested using logistic regression adjusted for age and sex for each HARE-assigned group, as well as for all groups combined.

Additional details can be found in the Supplemental Materials.

## Results

Between March 1, 2020, and February 2, 2021, we identified 19,168 (3.7%) COVID-19 positive cases among MVP participants, including 11,778 Whites, 4,893 Blacks, and 2,497 Hispanics, and 0.8% of MVP participants were hospitalized due to COVID-19 (Table 1). On average, COVID-19 positive MVP participants were slightly younger (58.7 years) compared with participants hospitalized with COVID-19 (63.4 years) and with population controls (60.4 years) (Supplementary Table S1). This population has been described in more detail elsewhere (Gaziano et al., 2021; Song et al., 2021).

**TABLE 1:** Descriptive statistics of MVP participants contributing to the association between genetic variants and COVID-19 outcomes.

	COVID-19 positive	COVID-19 hospitalized	Severe COVID-19	Population controls
N	19,168	4,234	947	4,92,854
Population				
White	11,778	2,417	543	3,57,198
Black	4,893	1,300	284	94,556
Hispanic	2,497	517	120	41,100
Age	58.7 ± 13.5	63.4 ± 11.4	66.7 ± 10.4	60.4 ± 13.7
Male	17,151 (89%)	3,976 (94%)	913 (96%)	4,44,753 (90%)

We performed genome-wide association for four COVID-19 outcomes (Supplementary Figure S1, S2): 1) COVID-19 positivity as defined by positive COVID-19 test compared with all other MVP participants (POS *vs*. POP); 2) individuals who were hospitalized for COVID-19 compared with all other MVP participants, including individuals who tested positive for COVID-19 but were not hospitalized (HOS *vs*. POP); 3) individuals who were hospitalized for COVID-19 compared with individuals who tested positive for COVID-19 but were not hospitalized (HOS *vs*. NOT); and 4) individuals who were hospitalized for COVID-19 with high-flow oxygen or died of COVID-19 (severe COVID-19) compared with all other MVP participants (SEV *vs*. POP).

**FIGURE 1:**
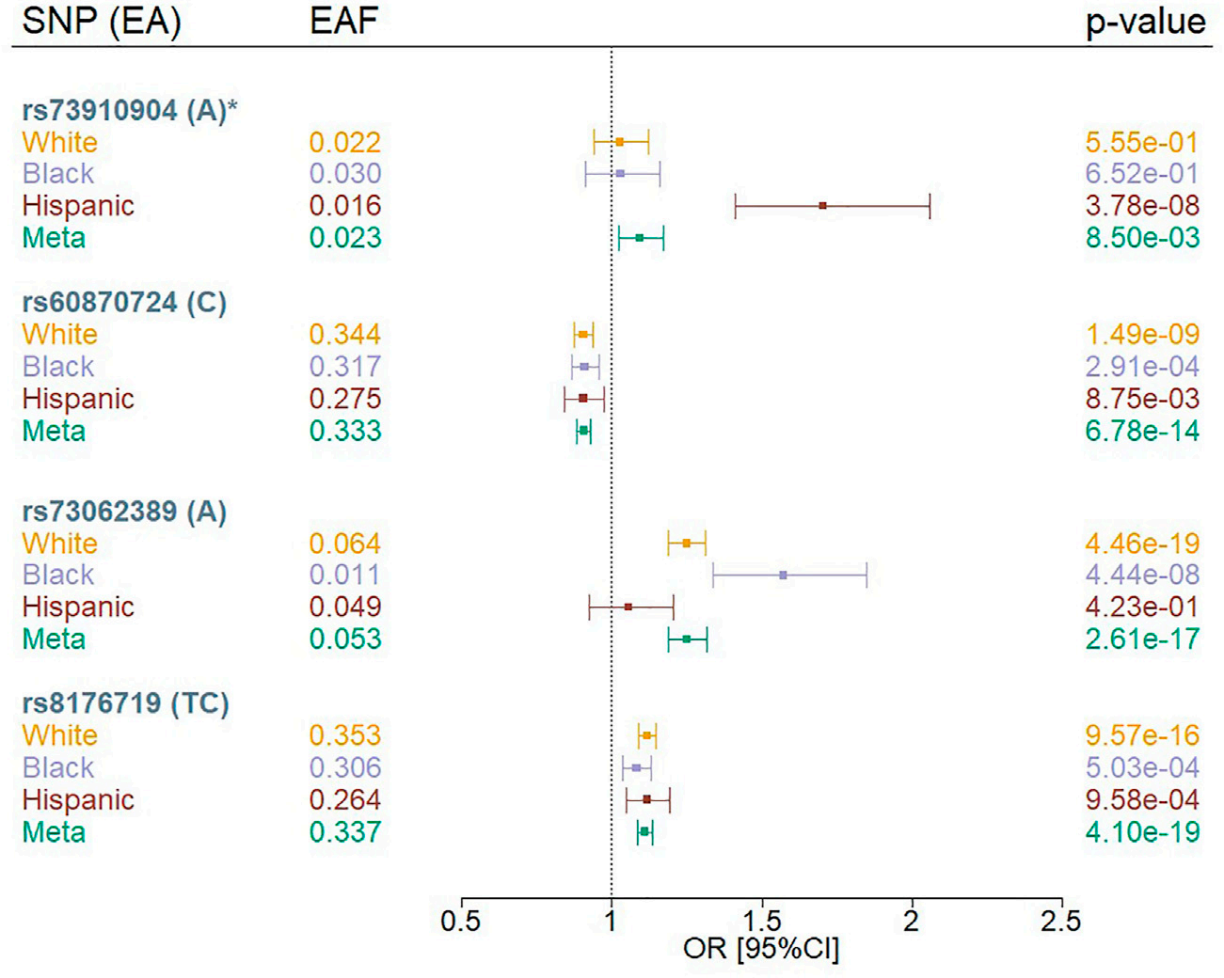
Forest plots of SNPs associated with COVID-19 positivity (POS *vs*. POP) in MVP by population. EA = effect allele, EAF = effect allele frequency, OR = odds ratio; Meta = Multi-population meta-analysis * indicates that the SNP association was novel.

We identified four independent variants that met a genome-wide significance threshold (p < 5 × 10^−08^) in the multi-population meta-analysis or population-specific analysis using the MVP COVID-19 positivity outcome (POS *vs*. POP) (Figure 1; Supplementary Table S2). The four variants were rs73910904 on chromosome 2, rs73062389 and rs60870724 on chromosome 3, and rs8176719 on chromosome 9. We did not observe any genome-wide significant associations for the MVP COVID-19 hospitalization outcomes.

Three of our genome-wide significant variants (rs73062389, rs8176719, and rs60870724) were in loci previously reported to be associated with COVID-19 phenotypes among Whites (Severe Covid-19 GWAS Group et al., 2020; COVID-19 Host Genetics Initiative, 2021; Pairo-Castineira et al., 2021) (Figure 1, Supplementary Table S2). The previously reported chromosome 3 locus at rs73062389 was also associated at genome-wide significance in Blacks. We observed that rs8176719 on chromosome 9 was in high linkage disequilibrium (LD) with the previously reported variant, rs657152 (Severe Covid-19 GWAS Group et al., 2020) (r^2^ = 0.97), but rs73062389 was not in LD with the previously reported SNPs, rs11385942 (Severe Covid-19 GWAS Group et al., 2020), and rs73064425 (Pairo-Castineira et al., 2021) (r^2^ = 0) using a cosmopolitan 1000G reference panel.

We observed an association between rs73910904 (MAF = 2%) and COVID-19 positivity only in Hispanics, with each additional A allele contributing 1.7 increased odds of having a positive COVID-19 test compared to population controls (*p* = 3.8 × 10^−08^) (Supplementary Figure S3). The association appears to be population-specific despite similar allele frequencies across groups. This finding did not replicate in the COVID-19 Host Genetics Initiative (HGI) results (*p* < 0.05) using any of the HGI-defined outcomes (Supplementary Table S3). Furthermore, this variant maps to a long interspersed nuclear element (LINE; *L1MB7*) but does not overlap a structural variant reference panel (Collins et al., 2020).

While genetic effects were found to be in the same direction across White, Black, and Hispanic Veterans (Figure 1), some associations showed evidence of heterogeneity across HARE groups (Supplementary Table S2). The two common variants (rs60870724 and rs8176719) achieved nominal significance (*p* < 0.05) in all strata, but this was not the case for the rarer variants (rs73062389 and rs73910904). For the two common variants, we also observed odds ratios that diminished as the severity and specificity of the outcome increased (POS *vs*. POP > HOS *vs*. POP > HOS *vs*. NOT>SEV *vs*. POP) (Supplementary Figure S4). For example, the two common variants achieved nominal significance (*p* < 0.05) in the HOS *versus* POP analysis, but not in the HOS *versus* NOT analysis, likely due to the limited sample size in the HOS *versus* NOT analysis. For the less common variant, rs73062389, we observed a similar pattern; however, the odds ratio was not consistent in its effect across outcomes. The Hispanic-only association observed at rs73910904 for the COVID-19 susceptibility outcome was not observed in either analysis involving hospitalized MVP participants.

Gene-based analyses identified five significant gene associations with COVID-19 positivity on chromosome 3 (*p* < 0.05/19,148 genes = 2.6 × 10^−06^): *NXPE3* (*p* = 1.6 × 10^−11^), *ZBTB11* (*p* = 5.8 × 10^−11^), *CEP97* (*p* = 1.0 × 10^−10^), *RPL24I* (*p* = 9.4 × 10^−09^), and *PCNP* (*p* = 6.8 × 10^−07^). The significant genes were located in a 682 kb region around the chromosome 3 locus indexed by lead SNP rs60870724. This region spanned base pairs 100870610–101552553 of chromosome 3, including nine other significant independent SNPs and 233 other SNPs in LD (r^2^ > 0.6) with the significant SNPs. There were no other significant gene associations with any of the three COVID-19 phenotypes. The other chromosome 3 locus (bps 45637109–45839176) indexed by lead SNP rs73062389 (*p* = 2.6 × 10^−17^) contained a suggestive but non-significant association between *SLC6A20* and COVID-19 positivity (*p* = 4.8 × 10^−05^). The ten strongest gene-phenotype associations are presented in Supplementary Table S4. A Manhattan plot of gene associations with COVID-19 positivity is presented in Supplementary Figure S5.

Gene-set analyses identified a single significant association (*p* < 0.05/19,148 gene sets = 2.6 × 10^−06^) between the gene set for regulation of oogenesis (N_genes_ = 10) and COVID-19 hospitalization (*p* = 1.4 × 10^−07^). No single gene in the gene-set was significantly associated with COVID-19 hospitalization in the single-gene tests. However, *PDE3A* was nominally associated with COVID-19 positivity (*p* = 0.019). The top 10 gene-set associations for each trait are presented in Supplementary Table S5.

Next, we determined whether the previously reported COVID-19 genetic associations were associated with MVP-defined COVID-19 outcomes. We replicated eight out of nine previously reported genetic associations (Severe Covid-19 GWAS Group et al., 2020; Pairo-Castineira et al., 2021) at an alpha of 0.05 (Supplementary Table S6) in at least one population-specific or multi-population meta-analysis for one of the four MVP COVID-19 outcomes.

**FIGURE 2:**
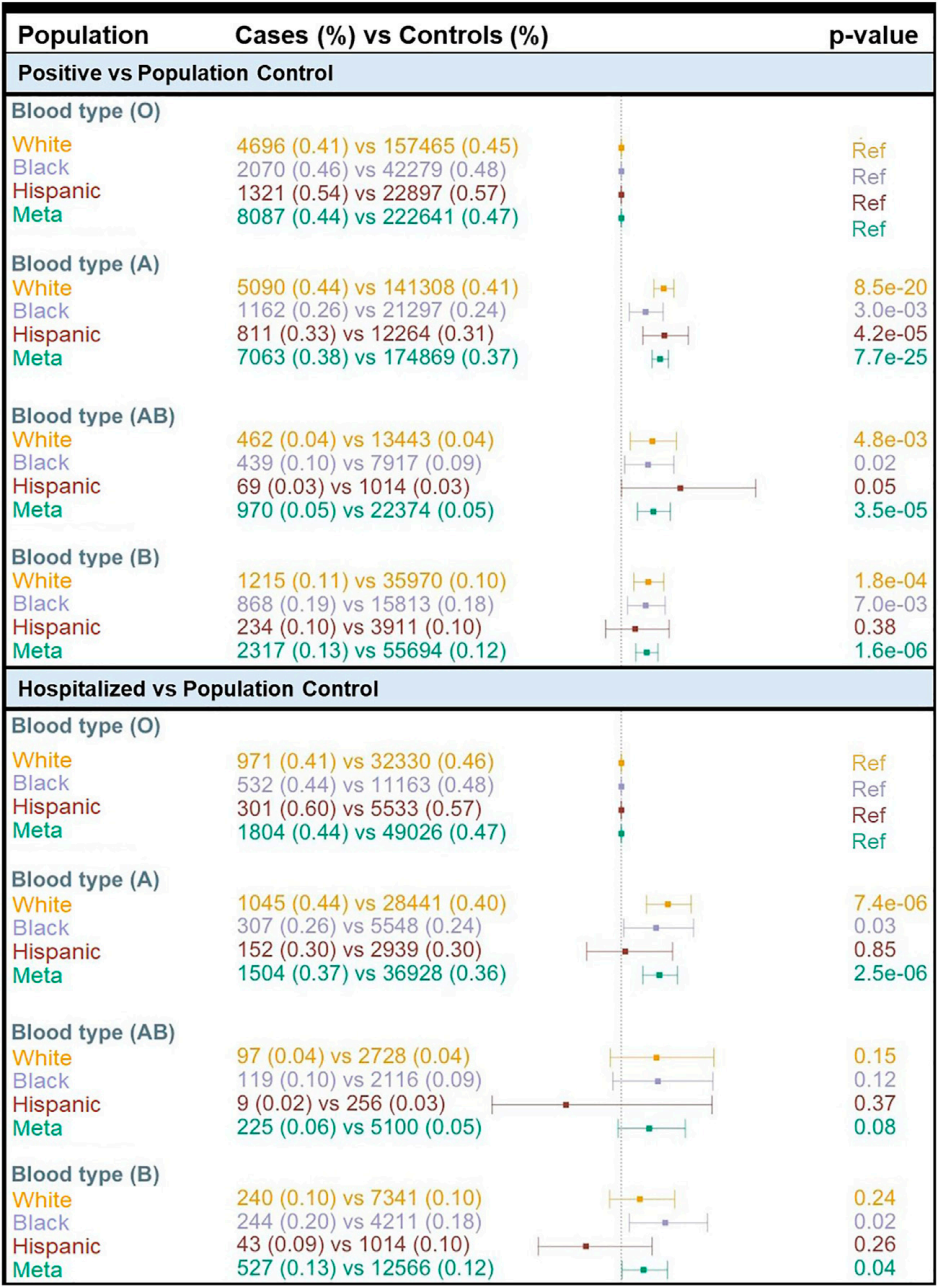
Association of COVID-19 with ABO blood type by population. OR = odds ratio; Meta = Multi-population meta-analysis; Ref = Reference.

We found a concordance of 99% between ABO blood group assignment using genotypes and serology typing (Supplementary Table S7). Consistent with the prevalence of the ABO blood group in the general population based on serology, we found that 47% of MVP participants had O inferred blood type while 32.7, 15.0, and 5.3% had A, B, and AB inferred blood types, respectively (Supplementary Table S7). We compared COVID-19 susceptibility between blood types using O as a reference within each population and among all individuals combined and found that A, AB, and B blood types were significantly associated with higher COVID-19 susceptibility (POS *vs*. POP) when compared with O blood type (Figure 2). The effect size was greater for A blood type (OR = 1.19; 95% CI: 1.15–1.23; *p* = 7.7 × 10^−25^) compared to AB and B blood groups (Figure 2; Supplementary Table S8). We observed similar results when restricting to only participants tested for COVID-19 (Supplementary Table S8). When considering the MVP hospitalization outcome, we observed that having the A blood type was associated with a higher risk of hospitalization among Whites (OR = 1.23; 95% CI: 1.12–1.35; *p* = 7.4 × 10^−06^) and Blacks (OR = 1.17; 95% CI: 1.10–1.35; *p* = 0.03) (Supplementary Table S8). A similar association was not observed among Hispanics, likely due to the lower number of cases in this population.

## Discussion

We utilized the MVP to identify four genetic variants significantly associated with COVID-19 infection in multi-population analyses of White, Black, and Hispanic Veterans. Three of these SNPs are located in genomic regions implicated in published COVID-19 studies, including rs73062389 and rs8176719 in the Severe Covid GWAS Group Study (Severe Covid-19 GWAS Group et al., 2020) and rs60870724 in HGI (COVID-19 Host Genetics Initiative, 2021).

The novel rs73910904 SNP was significantly associated with COVID-19 infection only among Hispanic Veterans. Further analyses showed that 1) the same SNP showed no effect in other ancestry groups in the MVP despite their higher allele frequencies and greater sample sizes and 2) rs73910904 did not replicate in a follow-up HGI (COVID-19 Host Genetics Initiative, 2021) query. Collectively, these data suggest that the rs73910904 association may be a false positive. The lack of replication could be attributable to statistical power but is unlikely due to the size of HGI. Hispanic Veterans in MVP have varying proportions of admixture from different ancestry groups due to their population history. Therefore, this heterogeneity may be a likely explanation for the lack of replication.

The most well-known SNP commonly used to infer genetically derived blood type, rs8176719, was similarly associated with COVID-19 positivity across populations. Given this association, we inferred ABO blood type for all MVP participants and found all non-O blood types were associated with COVID-19 infection (both positive *vs*. population and positive *vs*. negative tests) compared with the O blood type, and the A blood type was also associated with hospitalization in Whites and Blacks.

Blood types have previously been associated with 11 cardiovascular health indices, including thrombosis, hypertension, heart failure, and atherosclerosis (Groot et al., 2020). Differences in risk between A and B for these cardiovascular indices were mostly nonsignificant in the UK Biobank (UKBB). This may suggest that the critical distinction is between O and non-O blood types (Groot et al., 2020). The pattern observed in the UKBB is further substantiated in this report in MVP since the genome-wide significant rs8176719 is the key locus for distinguishing O type blood from A and B (Paterson et al., 2009). We are unaware of other studies that had adequate power to examine the AB blood type with medical outcomes, underscoring the unique contribution of our findings. Moreover, we are unaware of other published data with sufficient sample size to examine blood type associations with COVID-19 phenotypes in non-European ancestry groups. The differential risk for COVID-19 susceptibility by blood type alone is an area of active investigation and may not be fully explained by differences in cardiovascular health. Individuals with non-O blood types in general and blood types A and B specifically may experience higher susceptibility to COVID-19 infection due to poorer cardiovascular health at baseline than O type individuals (Dai, 2020). Healthcare use associated with greater cardiovascular disease risk at baseline may also explain seeking medical care, COVID-19 testing, and COVID-19 positivity. On the contrary, the association between blood type and COVID-19 infection may result from pleiotropic effects of the rs8176719 SNP, as this variant is implicated in several biological mechanisms (Verbanck et al., 2018). One such example is that the same ABO antigen-related glycans expressed in red blood cells are also expressed on the surface of the respiratory epithelial cells with slight modifications, which may render a preferential binding of blood type A glycans to the SARS-CoV-2 (Wu et al., 2021) explaining in part the increased susceptibility to COVID-19 infection. However, these results are still partially inconsistent with our findings because we found increased COVID-19 infection risk not only in blood group A but also in groups B and AB. Future molecular work will be needed to investigate potential mechanisms underpinning this associated risk.

Four genes near our chromosome 3 locus—*NXPE3*, *ZBTB11*, *CEP97*, and *RPL24I—*have been linked to COVID-19 SNPs in HGI (COVID-19 Host Genetics Initiative, 2021) and studies based on HGI data (Rao et al., 2021). No studies to date have identified which genes in the region are probable therapeutic targets. Our lead SNP for this region in MVP was rs60870724, an intergenic indel located 2.7 kb upstream of *NXPE3* and 52.6 kb downstream of *CEP97*. The proximity of rs60870724 to *NXPE3*, combined with the strength of the gene-based association, points to *NXPE3* as a potential causal gene, but further investigation is required to untangle the role of these genes in COVID-19. Our gene-based analyses also support the involvement of *SLC6A20* in COVID-19 (*p* = 4.8 × 10^−05^). Several SNPs from this gene have been previously reported as associated with COVID-19 positivity in HGI (COVID-19 Host Genetics Initiative, 2021), although with a different lead SNP: rs2271616. Previous functional experiments on variants in the 3p21.31 locus have also confirmed that *SLC6A20* and neighboring gene *CCR9* are the most likely causal genes in the region (Yao et al., 2021). In the experimental study, Yao et al. used CRISPR/Cas9 in COVID-19 relevant cell types to delete a 67.8 kb region containing 22 potential causal variants from 3p21.31. The authors found that gene expression of *SLC6A20* and *CCR9* changed more than other genes in response to genome editing.

We found that the gene set for GO biological process “regulation of oogenesis” was significantly associated with COVID-19 hospitalization (*p* = 1.4 × 10^−07^). The genes responsible for this association were likely *PDE3A*, which had a nominal association with COVID-19 positivity (*p* = 0.018), and *IGF1*, for which we were underpowered to detect an association with COVID-19 hospitalization in our study (*p* = 0.14) but which has been implicated in previous COVID-19 studies (Fan et al., 2021). A small case report of PDE3-inhibitor enoximone found substantial benefit for four patients with respiratory failure from SARS-CoV-2 pneumonia compared to three control patients treated with standard care (Beute et al., 2021). Patients treated with the PDE3-inhibitor experienced symptomatic relief within a few hours (N = 2) or within 24–36 h (N = 2) and were able to forgo mechanical ventilation. Insulin-like growth factor (IGF-1) was previously found associated with COVID-19 mortality in UKBB (Fan et al., 2021). The authors found each 1-standard deviation increase in log-transformed IGF-1 reduced odds of COVID-19 mortality by 15% (OR = 0.85, 95% CI: 0.73–0.99). Furthermore, UKBB patients in the highest quartile of IGF-1 had a 41% lower risk for COVID-19 mortality than patients in the lowest quartile. Similar trends persisted across stratified and sensitivity analyses. The significant association between the gene set containing these genes and COVID-19 hospitalization suggests there may be a genetic basis for the previously reported associations between protein levels of PDE3A and IGF-1 with severe COVID-19. Further investigation of pQTLs in *PDE3A* and *IGF-1* will be needed to confirm the genetic relationship between these two proteins and COVID-19.

The finding that eight out of the nine early SNP associations were replicated at nominal *P* value significance of <0.05 in the MVP is an important addition to the literature. Early studies had limited power for genome-wide analyses and focused on severe COVID-19 phenotypes. Specifically, the rs657152 SNP was associated with respiratory failure in those with COVID-19 (Severe Covid-19 GWAS Group et al., 2020), and eight additional SNPs were associated with life-threatening COVID-19 (Pairo-Castineira et al., 2021). The fact that five of these SNPs were associated with MVP COVID-19 infection is intriguing as it might have been assumed that the most significantly associated SNPs in the earlier studies conferred a risk for a severe reaction to COVID-19 rather than a risk for a COVID-19 infection. As an increasing proportion of the population is exposed to the virus, with larger numbers of severe cases, other host genetic variations may yet be identified that predict a more severe course of the illness. Another contribution of this work is the opportunity to examine genetic associations across populations defined by genetic ancestry and self-reported race/ethnicity. Even with limited power to examine SNP effects in population groups, there are clear examples of general, multi-population effects (e.g., rs73064425 shows an effect across Whites, Blacks, and Hispanics) and examples of effects limited to participants from the largest group (e.g., SNPs rs143334143, rs21009069, and rs9380142 in Whites). While the absence of an association in any subgroup may be related to power limitations, the genetic variants with multi-population associations demonstrate the critically important contribution of the MVP towards extending genomic studies beyond one group.

We acknowledge limitations, including limited power for genome-wide analyses in our severity COVID-19 outcomes. Given our 947 cases with severe COVID-19, we only reach >90% power if our genotypic relative risk >1.35 with a 50% frequency variant. Power is substantially reduced for lower genotypic relative risks or lower frequency variants. Overall, none of the SNPs achieved genome-wide significance for hospitalization outcomes, likely attributable to the small number of patients who were hospitalized within VA hospitals. The HGI may aggregate data across sites to address this shortcoming in future analyses of severe COVID-19 infection. Similarly, we did not examine death as an isolated outcome given the limited number of accrued deaths at the time of the analysis. Nevertheless, the power obtained within the MVP compares favorably with published efforts to date (particularly in people of non-European ancestry). Another inherent limitation to the MVP dataset is the predominance of males given the nature of the Veteran population, though efforts continue to oversample female Veterans. Additionally, we could only capture those participants in the MVP who came to the VA for care or had their COVID-19 status reported to VA. The incomplete capture of relevant clinical data compounded by the lack of uniform, general SARS-CoV-2 testing in the United States possibly impacted the statistical power for analyses that used the whole non-COVID-19 positive MVP population as controls, which may have included asymptomatic carriers who were not tested. This misclassification bias may have led to underestimating the true effect sizes of the associations. It is also important to note that potential differences in SARS-CoV-2 variants were not accounted for in these analyses. Specifically, at least the Alpha and Beta variants (formerly known as B.1.1.7 and B.1.351, resp.) were present in the United States during the timeframe assessed in this study and may have infected Veterans included in these analyses. Any differences in the genetics of host vulnerability (or resistance) to morbidity and mortality to such COVID-19 variants are not able to be accounted for as SARS-CoV-2 variant testing is not routinely performed in this population or other US populations (Crawford and Williams, 2021). We are also unable to discount that utilization bias may have differentially impacted who was tested for SARS-CoV-2. Finally, while vaccination status may have impacted the likelihood of infection, doses were not administered in the VA until January 2021, and case status was determined between March 1, 2020, and February 2, 2021. Therefore, the impact on the findings will be minimal. These dates are also prior to the Delta variant sweep in the United States. Despite these limitations, we identified SNPs associated with COVID-19 outcomes that are consistent with earlier reports of mostly White individuals (Severe Covid-19 GWAS Group et al., 2020; Pairo-Castineira et al., 2021) and were able to replicate them across Blacks and Hispanics, in addition to reporting a new genetic association that requires further investigation.

## Data Availability

The datasets presented in this study can be found in online repositories. The names of the repository can be found below: Full GWAS summary statistics can be found in dbGaP (https://www.ncbi.nlm.nih.gov/gap/) under the MVP accession (phs001672).

## References

[B1] Arons M. M. Hatfield K. M. Reddy S. C. Kimball A. James A. Jacobs J. R. (2020). Presymptomatic SARS-CoV-2 Infections and Transmission in a Skilled Nursing Facility. N. Engl. J. Med. 382, 2081–2090. 10.1056/NEJMoa2008457 32329971 PMC7200056

[B2] Beute J. Boermans P. Benraad B. Telman J. Diamant Z. KleinJan A. (2021). PDE3-inhibitor Enoximone Prevented Mechanical Ventilation in Patients with SARS-CoV-2 Pneumonia. Exp. Lung Res. 47, 1–12. 10.1080/01902148.2021.1881189 33544007 PMC7876671

[B3] Byambasuren O. Cardona M. Bell K. Clark J. McLaws M.-L. Glasziou P. (2020). Estimating the Extent of Asymptomatic COVID-19 and its Potential for Community Transmission: Systematic Review and Meta-Analysis. Official J. Assoc. Med. Microbiol. Infect. Dis. Can. 5, 223–234. 10.3138/jammi-2020-0030 PMC9602871 36340059

[B4] Chang C. C. Chow C. C. Tellier L. C. Vattikuti S. Purcell S. M. Lee J. J. (2015). Second-generation PLINK: Rising to the challenge of Larger and Richer Datasets. GigaSci 4, 7. 10.1186/s13742-015-0047-8 PMC4342193 25722852

[B5] Chapman A. Peterson K. Turano A. Box T. Wallace K. Jones M. (2020). “A Natural Language Processing System for National COVID-19 Surveillance in the US Department of Veterans Affairs,” in Proceedings of the 1st Workshop on NLP for COVID-19, July 5-10, 2020 (Association for Computational Linguistics).

[B6] Collins R. L. Brand H. Karczewski K. J. Zhao X. Alföldi J. Francioli L. C. (2020). A Structural Variation Reference for Medical and Population Genetics. Nature 581, 444–451. 10.1038/s41586-020-2287-8 32461652 PMC7334194

[B7] COVID-19 Host Genetics Initiative (2021). Mapping the Human Genetic Architecture of COVID-19. Nature 600, 472–477. 10.1038/s41586-021-03767-x 34237774 PMC8674144

[B8] Crawford D. C. Williams S. M. (2021). Global Variation in Sequencing Impedes SARS-CoV-2 Surveillance. Plos Genet. 17, e1009620. 10.1371/journal.pgen.1009620 34264957 PMC8282079

[B9] Dai X. (2020). ABO Blood Group Predisposes to COVID-19 Severity and Cardiovascular Diseases. Eur. J. Prev. Cardiolog 27, 1436–1437. 10.1177/2047487320922370 PMC7717262 32343152

[B10] de Leeuw C. A. Mooij J. M. Heskes T. Posthuma D. (2015). MAGMA: Generalized Gene-Set Analysis of GWAS Data. Plos Comput. Biol. 11, e1004219. 10.1371/journal.pcbi.1004219 25885710 PMC4401657

[B11] de Leeuw C. A. Stringer S. Dekkers I. A. Heskes T. Posthuma D. (2018). Conditional and Interaction Gene-Set Analysis Reveals Novel Functional Pathways for Blood Pressure. Nat. Commun. 9, 3768. 10.1038/s41467-018-06022-6 30218068 PMC6138636

[B12] Dean M. Carrington M. Winkler C. Huttley G. A. Smith M. W. Allikmets R. (1996). Genetic Restriction of HIV-1 Infection and Progression to AIDS by a Deletion Allele of the CKR5 Structural Gene. Science 273, 1856–1862. 10.1126/science.273.5283.1856 8791590

[B13] Severe Covid-19 GWAS Group Ellinghaus D. Degenhardt F. Bujanda L. Buti M. Albillos A. Invernizzi P (2020). Genomewide Association Study of Severe Covid-19 with Respiratory Failure. N. Engl. J. Med. 383, 1522–1534. 10.1056/NEJMoa2020283 32558485 PMC7315890

[B14] Fan X. Yin C. Wang J. Yang M. Ma H. Jin G. (2021). Pre-diagnostic Circulating Concentrations of Insulin-like Growth Factor-1 and Risk of COVID-19 Mortality: Results from UK Biobank. Eur. J. Epidemiol. 36, 311–318. 10.1007/s10654-020-00709-1 33420872 PMC7794621

[B15] Fang H. Hui Q. Lynch J. Honerlaw J. Assimes T. L. Huang J. (2019). Harmonizing Genetic Ancestry and Self-Identified Race/Ethnicity in Genome-wide Association Studies. Am. J. Hum. Genet. 105, 763–772. 10.1016/j.ajhg.2019.08.012 31564439 PMC6817526

[B16] Garg S. Kim L. Whitaker M. O’Halloran A. Cummings C. Holstein R. (2020). Hospitalization Rates and Characteristics of Patients Hospitalized with Laboratory-Confirmed Coronavirus Disease 2019 - COVID-NET, 14 States, March 1-30, 2020. MMWR Morb. Mortal. Wkly. Rep. 69, 458–464. 10.15585/mmwr.mm6915e3 32298251 PMC7755063

[B17] Gaziano J. M. Concato J. Brophy M. Fiore L. Pyarajan S. Breeling J. (2016). Million Veteran Program: A Mega-Biobank to Study Genetic Influences on Health and Disease. J. Clin. Epidemiol. 70, 214–223. 10.1016/j.jclinepi.2015.09.016 26441289

[B18] Gaziano L. Giambartolomei C. Giambartolomei C. Pereira A. C. Gaulton A. Posner D. C. (2021). Actionable Druggable Genome-wide Mendelian Randomization Identifies Repurposing Opportunities for COVID-19. Nat. Med. 27, 668–676. 10.1038/s41591-021-01310-z 33837377 PMC7612986

[B19] Goyal P. Choi J. J. Pinheiro L. C. Schenck E. J. Chen R. Jabri A. (2020). Clinical Characteristics of Covid-19 in New York City. N. Engl. J. Med. 382, 2372–2374. 10.1056/NEJMc2010419 32302078 PMC7182018

[B20] Grasselli G. Zangrillo A. Zanella A. Antonelli M. Cabrini L. Castelli A. (2020). Baseline Characteristics and Outcomes of 1591 Patients Infected with SARS-CoV-2 Admitted to ICUs of the Lombardy Region, Italy. JAMA 323, 1574–1581. 10.1001/jama.2020.5394 32250385 PMC7136855

[B21] Groot H. E. Villegas Sierra L. E. Said M. A. Lipsic E. Karper J. C. van der Harst P. (2020). Genetically Determined ABO Blood Group and its Associations with Health and Disease. Atvb 40, 830–838. 10.1161/ATVBAHA.119.313658 31969017

[B22] Gu T. Mack J. A. Salvatore M. Prabhu Sankar S. Valley T. S. Singh K. (2020). Characteristics Associated with Racial/Ethnic Disparities in COVID-19 Outcomes in an Academic Health Care System. JAMA Netw. Open 3, e2025197. 10.1001/jamanetworkopen.2020.25197 33084902 PMC7578774

[B23] Hunter-Zinck H. Shi Y. Li M. Gorman B. R. Ji S.-G. Sun N. (2020). Genotyping Array Design and Data Quality Control in the Million Veteran Program. Am. J. Hum. Genet. 106, 535–548. 10.1016/j.ajhg.2020.03.004 32243820 PMC7118558

[B24] Ioannou G. N. Locke E. Green P. Berry K. O’Hare A. M. Shah J. A. (2020). Risk Factors for Hospitalization, Mechanical Ventilation, or Death Among 10 131 US Veterans with SARS-CoV-2 Infection. JAMA Netw. Open 3, e2022310. 10.1001/jamanetworkopen.2020.22310 32965502 PMC7512055

[B25] Khor C. C. Hibberd M. L. (2012). Host-pathogen Interactions Revealed by Human Genome-wide Surveys. Trends Genet. 28, 233–243. 10.1016/j.tig.2012.02.001 22445588

[B26] Klarin D. Damrauer S. M. Damrauer S. M. Cho K. Sun Y. V. Teslovich T. M. (2018). Genetics of Blood Lipids Among ∼300,000 Multi-Ethnic Participants of the Million Veteran Program. Nat. Genet. 50, 1514–1523. 10.1038/s41588-018-0222-9 30275531 PMC6521726

[B27] Kwok A. J. Mentzer A. Knight J. C. (2021). Host Genetics and Infectious Disease: New Tools, Insights and Translational Opportunities. Nat. Rev. Genet. 22, 137–153. 10.1038/s41576-020-00297-6 33277640 PMC7716795

[B28] Mägi R. Morris A. P. (2010). GWAMA: Software for Genome-wide Association Meta-Analysis. BMC Bioinformatics 11, 288. 10.1186/1471-2105-11-288 20509871 PMC2893603

[B29] Manichaikul A. Mychaleckyj J. C. Rich S. S. Daly K. Sale M. Chen W.-M. (2010). Robust Relationship Inference in Genome-wide Association Studies. Bioinformatics 26, 2867–2873. 10.1093/bioinformatics/btq559 20926424 PMC3025716

[B30] Oran D. P. Topol E. J. (2021). Prevalence of Asymptomatic SARS-CoV-2 Infection. Ann. Intern. Med. 174, 286–287. 10.7326/L20-1285 33587872

[B31] Pairo-Castineira E. Clohisey S. Clohisey S. Klaric L. Bretherick A. D. Rawlik K. (2021). Genetic Mechanisms of Critical Illness in COVID-19. Nature 591, 92–98. 10.1038/s41586-020-03065-y 33307546

[B32] Paré G. Chasman D. I. Kellogg M. Zee R. Y. L. Rifai N. Badola S. (2008). Novel Association of ABO Histo-Blood Group Antigen with Soluble ICAM-1: Results of a Genome-wide Association Study of 6,578 Women. Plos Genet. 4, e1000118. 10.1371/journal.pgen.1000118 18604267 PMC2432033

[B33] Paterson A. D. Lopes-Virella M. F. Waggott D. Boright A. P. Hosseini S. M. Carter R. E. (2009). Genome-wide Association Identifies the ABO Blood Group as a Major Locus Associated with Serum Levels of Soluble E-Selectin. Atvb 29, 1958–1967. 10.1161/ATVBAHA.109.192971 PMC3147250 19729612

[B34] Petrilli C. M. Jones S. A. Yang J. Rajagopalan H. O’Donnell L. Chernyak Y. (2020). Factors Associated with Hospital Admission and Critical Illness Among 5279 People with Coronavirus Disease 2019 in New York City: Prospective Cohort Study. BMJ 369, m1966. 10.1136/bmj.m1966 32444366 PMC7243801

[B35] Price A. L. Patterson N. J. Plenge R. M. Weinblatt M. E. Shadick N. A. Reich D. (2006). Principal Components Analysis Corrects for Stratification in Genome-wide Association Studies. Nat. Genet. 38, 904–909. 10.1038/ng1847 16862161

[B36] Rao S. Baranova A. Cao H. Chen J. Zhang X. Zhang F. (2021). Genetic Mechanisms of COVID-19 and its Association with Smoking and Alcohol Consumption. Brief Bioinform 22, bbab284. 10.1093/bib/bbab284 34308962

[B37] Samson M. Libert F. Doranz B. J. Rucker J. Liesnard C. Farber C.-M. (1996). Resistance to HIV-1 Infection in Caucasian Individuals Bearing Mutant Alleles of the CCR-5 Chemokine Receptor Gene. Nature 382, 722–725. 10.1038/382722a0 8751444

[B38] Song R. J. Ho Y.-L. Schubert P. Park Y. Posner D. Lord E. M. (2021). Phenome-wide Association of 1809 Phenotypes and COVID-19 Disease Progression in the Veterans Health Administration Million Veteran Program. PLoS One 16, e0251651. 10.1371/journal.pone.0251651 33984066 PMC8118298

[B39] Sutton D. Fuchs K. D’Alton M. Goffman D. (2020). Universal Screening for SARS-CoV-2 in Women Admitted for Delivery. N. Engl. J. Med. 382, 2163–2164. 10.1056/NEJMc2009316 32283004 PMC7175422

[B40] Sze S. Pan D. Nevill C. R. Gray L. J. Martin C. A. Nazareth J. (2020). Ethnicity and Clinical Outcomes in COVID-19: A Systematic Review and Meta-Analysis. EClinicalMedicine 29-30, 100630. 10.1016/j.eclinm.2020.100630 PMC7658622 33200120

[B41] Vahidy F. S. Nicolas J. C. Meeks J. R. Khan O. Pan A. Jones S. L. (2020). Racial and Ethnic Disparities in SARS-CoV-2 Pandemic: Analysis of a COVID-19 Observational Registry for a Diverse US Metropolitan Population. BMJ Open 10, e039849. 10.1136/bmjopen-2020-039849 PMC7418666 32784264

[B42] Verbanck M. Chen C.-Y. Neale B. Do R. (2018). Detection of Widespread Horizontal Pleiotropy in Causal Relationships Inferred from Mendelian Randomization between Complex Traits and Diseases. Nat. Genet. 50, 693–698. 10.1038/s41588-018-0099-7 29686387 PMC6083837

[B43] Watanabe K. Taskesen E. van Bochoven A. Posthuma D. (2017). Functional Mapping and Annotation of Genetic Associations with FUMA. Nat. Commun. 8, 1826. 10.1038/s41467-017-01261-5 29184056 PMC5705698

[B44] Webb Hooper M. Nápoles A. M. Pérez-Stable E. J. (2020). COVID-19 and Racial/Ethnic Disparities. JAMA 323, 2466–2467. 10.1001/jama.2020.8598 32391864 PMC9310097

[B45] Wu C. Chen X. Cai Y. Xia J. a. Zhou X. Xu S. (2020). Risk Factors Associated with Acute Respiratory Distress Syndrome and Death in Patients with Coronavirus Disease 2019 Pneumonia in Wuhan, China. JAMA Intern. Med. 180, 934–943. 10.1001/jamainternmed.2020.0994 32167524 PMC7070509

[B46] Wu S.-C. Arthur C. M. Wang J. Verkerke H. Josephson C. D. Kalman D. (2021). The SARS-CoV-2 Receptor-Binding Domain Preferentially Recognizes Blood Group A. Blood Adv. 5, 1305–1309. 10.1182/bloodadvances.2020003259 33656534 PMC7929867

[B47] Yao Y. Ye F. Li K. Xu P. Tan W. Feng Q. (2021). Genome and Epigenome Editing Identify CCR9 and SLC6A20 as Target Genes at the 3p21.31 Locus Associated with Severe COVID-19. Sig Transduct Target. Ther. 6, 85. 10.1038/s41392-021-00519-1 PMC7897877 33619245

[B48] Zhou F. Yu T. Du R. Fan G. Liu Y. Liu Z. (2020). Clinical Course and Risk Factors for Mortality of Adult Inpatients with COVID-19 in Wuhan, China: a Retrospective Cohort Study. The Lancet 395, 1054–1062. 10.1016/S0140-6736(20)30566-3 PMC7270627 32171076

