# Supplementary material for: Genetic Loci Associated With COVID-19 Positivity and Hospitalization in White, Black, and Hispanic Veterans of the VA Million Veteran Program
Source: Front Genet. 2022 Feb 3;12:777076. doi: 10.3389/fgene.2021.777076 (PMC8864634; doi:10.3389/fgene.2021.777076)
Supplement: Supplementary file 2 [file DataSheet1.DOCX]

**Supplemental Materials**

1. COVID-19 GWAS Working Group Membership 2
2. VA Million Veteran Program COVID-19 Science Initiative Membership & Acknowledgements 3
3. VA Million Veteran Program Core Membership & Acknowledgments 4
4. Supplemental Methods 9
5. Supplemental Figures 12
6. Supplemental Tables 25

*additional Supplemental Tables in Excel Document

**MVP COVID-19 GWAS Working Group**

Co-Chairs: Jennifer E. Huffman^1^, Yan V. Sun^2,3^

Members: Mehrdad Arjomandi^4,5^, Themistocles L Assimes^6-8^, Kyong-Mi Chang^9,10^, Dana C. Crawford^11-13^, Sharvari Dalal^9,14^, Jimmy T. Efird^15^, Joel Gelernter^16,17^, Richard L. Hauger^18,19^, Yuk-Lam Ho^1^, Austin T Hilliard^6-8^, Rose D.L. Huang^1^, Adriana M. Hung^20,21^, Darshana Jhala^9,14^, Jacob Joseph^1,22,23^, Michael S Lewis^24,25^, Shiuh-Wen Luoh^26,27^, Merry-Lynn N. McDonald^28,29^, John E. McGeary^30,31^, Valerio Napolioni^32,33^, Pradeep Natarajan^1,34-36^, Christopher J. O’Donnell^1,22,23^, Gita A. Pathak^16,37^, Gina M. Peloso^1,38^, Jeffrey Petersen^13,14^, Renato Polimanti^16,37^, Daniel C. Posner^1^, Panos Roussos^39,40^, Ran Tao^20,41,42^, Catherine Tcheandjieu^6-8,43^, Reid F. Thompson^44,45^, Philip S. Tsao^6,7^, Anurag Verma^9,10^, Frank R. Wendt^16,37^, Wen-Chih Wu^30,46^, Jin J Zhou^47,48^

^1^Massachusetts Veterans Epidemiology Research and Information Center (MAVERIC), VA Boston Healthcare System, Boston, MA, USA; ^2^Atlanta VA Health Care System, Decatur, GA, USA; ^3^Department of Epidemiology, Emory University Rollins School of Public Health, Atlanta, GA, USA; ^4^Medical Service, San Francisco VA Medical Center, San Francisco, CA, USA; ^5^Department of Medicine, University of California, San Francisco, CA, USA; ^6^VA Palo Alto Healthcare System, Palo Alto, CA, USA; ^7^Division of Cardiovascular Medicine, Department of Medicine, Stanford University School of Medicine, CA, USA; ^8^Stanford Cardiovascular Institute, Stanford University, Stanford, CA, USA; ^9^Corporal Michael J. Crescenz Veteran Affairs Medical Center, Philadelphia, PA, USA; ^10^Department of Medicine, Perelman School of Medicine, University of Pennsylvania, Philadelphia, PA, USA; ^11^Louis Stokes Cleveland VA Medical Center, Cleveland, OH, USA; ^12^Departments of Population and Quantitative Health Sciences & Genetics and Genomes Sciences, Case Western Reserve University, Cleveland, OH, USA; ^13^Cleveland Institute for Computational Biology, Case Western Reserve University, Cleveland, OH, USA; ^14^Department of Pathology and Laboratory Medicine, University of Pennsylvania Perelman School of Medicine, Philadelphia, PA, USA; ^15^Cooperative Studies Program Epidemiology Center-Durham, Durham VA Health Care System, Durham, NC, USA; ^16^VA CT West Haven, CT, USA; ^17^Yale University School of Medicine, Departments of Psychiatry, Genetics, and Neuroscience, New Haven, CT, USA; ^18^Center of Excellence for Stress and Mental Health (CESAMH), VA San Diego Healthcare System, San Diego, CA, USA; ^19^Center for Behavioral Genetics of Aging, School of Medicine, University of California San Diego, La Jolla, CA, USA; ^20^Tennessee Valley Healthcare System, Nashville, TN, USA; ^21^Vanderbilt University Medical Center, Division of Nephrology and Hypertension, Vanderbilt Center for Kidney Disease and Integrated Program for Acute Kidney Injury Research, and Vanderbilt Precision Nephrology Program Nashville, Nashville, TN, USA; ^22^Cardiology Section, VA Boston Healthcare System, Boston, MA, USA; ^23^Department Medicine, Brigham & Women's Hospital, Boston, MA, USA; ^24^Department of Pathology and Laboratory Medicine, West Los Angeles Veterans Affairs Medical Center, Los Angeles, CA, USA; ^25^Department of Medicine and Pathology, Cedars Sinai Medical Center, Los Angeles, CA, USA; ^26^VA Portland Health Care System, Portland, OR, USA; ^27^Knight Cancer Institute, Oregon Health & Science University, Portland, OR, USA; ^28^Birmingham VA Health Care System, Birmingham, AL, USA; ^29^Division of Pulmonary, Allergy and Critical Care Medicine, Department of Medicine, University of Alabama at Birmingham, Birmingham, AL, USA; ^30^Providence VA Healthcare System, Providence, RI, USA; ^31^Department of Psychiatry and Human Behavior, Brown University, Providence, RI, USA; ^32^Department of Neurology and Neurological Sciences, Stanford University School of Medicine, Stanford, CA, USA; ^33^Genomic And Molecular Epidemiology (GAME) Lab., School of Biosciences and Veterinary Medicine, Camerino, Italy; ^34^Department of Medicine, Harvard Medical School, Boston, MA, USA; ^35^Cardiovascular Research Center, Massachusetts General Hospital, Boston, MA, USA; ^36^Program in Medical and Population Genetics, Broad Institute of Harvard and MIT, Cambridge, MA, USA; ^37^Department of Psychiatry, Yale University, New Haven, CT, USA; ^38^Department of Biostatistics, Boston University School of Public Health, Boston, MA, USA; ^39^MIRECC, James J. Peters VA Medical Center, Bronx, NY, USA; ^40^Department of Psychiatry, Department of Genetics and Genomic Sciences, Icahn School of Medicine at Mount Sinai, New York, NY, USA; ^41^Department of Biostatistics, Vanderbilt University Medical Center, Nashville, TN, USA; ^42^Vanderbilt Genetics Institute, Vanderbilt University Medical Center, Nashville, TN, USA; ^43^Department of Pediatric Cardiology Stanford University School of Medicine, Stanford, California, USA; ^44^Division of Hospital & Specialty Medicine, VA Portland Healthcare System, Portland, OR, USA; ^45^Department of Radiation Medicine, Oregon Health & Science University, Portland, OR, USA; ^46^Department of Medicine, Alpert Medical School, Brown University, Providence, RI, USA; ^47^Phoenix VA Health Care System, Phoenix, AZ, USA; ^48^Department of Epidemiology and Biostatistics, University of Arizona, Tucson, AZ, USA

**VA Million Veteran Program COVID-19 Science Initiative**

**MVP COVID-19 Science Program Steering Committee**

- Christopher J. O’Donnell, M.D., M.P.H. (Co-Chair)

VA Boston Healthcare System, 150 S. Huntington Avenue, Boston, MA 02130

- J. Michael Gaziano, M.D., M.P.H. (Co-Chair)

VA Boston Healthcare System, 150 S. Huntington Avenue, Boston, MA 02130

- Philip S. Tsao, Ph.D. (Co-Chair)

VA Palo Alto Health Care System, 3801 Miranda Avenue, Palo Alto, CA 94304

- Sumitra Muralidhar, Ph.D.

US Department of Veterans Affairs, 810 Vermont Avenue NW, Washington, DC 20420

- Jean Beckham, Ph.D.

Durham VA Medical Center, 508 Fulton Street, Durham, NC 27705

- Kyong-Mi Chang, M.D.

Philadelphia VA Medical Center, 3900 Woodland Avenue, Philadelphia, PA 19104

- Juan P. Casas, M.D., Ph.D.

VA Boston Healthcare System, 150 S. Huntington Avenue, Boston, MA 02130

- Kelly Cho, M.P.H., Ph.D.

VA Boston Healthcare System, 150 S. Huntington Avenue, Boston, MA 02130

- Saiju Pyarajan, Ph.D.

VA Boston Healthcare System, 150 S. Huntington Avenue, Boston, MA 02130

- Jennifer Huffman, Ph.D.

VA Boston Healthcare System, 150 S. Huntington Avenue, Boston, MA 02130

- Jennifer Moser, Ph.D.

US Department of Veterans Affairs, 810 Vermont Avenue NW, Washington, DC 20420

**MVP COVID-19 Science Program Steering Committee Support**

- Lauren Thomann, M.P.H. (P&P Committee Representative, Working Group Coordinator)

VA Boston Healthcare System, 150 S. Huntington Avenue, Boston, MA 02130

- Helene Garcon, M.D. (Program Coordinator, Working Group Coordinator)

VA Boston Healthcare System, 150 S. Huntington Avenue, Boston, MA 02130

- Nicole Kosik, M.P.H. (Working Group Coordinator)

VA Boston Healthcare System, 150 S. Huntington Avenue, Boston, MA 02130

**MVP COVID-19 Science Program Working Groups and Associated Chairs**

- COVID-19 Related PheWAS
  - Katherine Liao, M.D.

VA Boston Healthcare System, 150 S. Huntington Avenue, Boston, MA 02130

- - Scott Damrauer, M.D.

Philadelphia VA Medical Center, 3900 Woodland Avenue, Philadelphia, PA 19104

- Disease Mechanisms
  - Richard Hauger, M.D.

VA San Diego Healthcare System, 3350 La Jolla Village Drive, San Diego, CA 92161

- - Shiuh-Wen Luoh, M.D., Ph.D.

Portland VA Medical Center, 3710 SW U.S. Veterans Hospital Road, Portland, OR 97239

- - Sudha Iyengar, Ph.D.

VA Northeast Ohio Healthcare System, 10701 East Boulevard, Cleveland, OH 44106

- Druggable Genome
  - Juan P. Casas, M.D., Ph.D.

VA Boston Healthcare System, 150 S. Huntington Avenue, Boston, MA 02130

- Genomics for Risk Prediction, PRS, and MR
  - Themistocles Assimes, M.D., Ph.D.

VA Palo Alto Health Care System, 3801 Miranda Avenue, Palo Alto, CA 94304

- - Panagiotis Roussos, M.D., Ph.D.

James J. Peters VA Medical Center, [130 W Kingsbridge Rd, Bronx, NY 10468](https://www.bing.com/local?lid=YN873x12457663610017047750&id=YN873x12457663610017047750&q=Emergency+Dept%2c+James+J+Peters+VA+Hospital&name=Emergency+Dept%2c+James+J+Peters+VA+Hospital&cp=40.86751174926758%7e-73.9051284790039&ppois=40.86751174926758_-73.9051284790039_Emergency+Dept%2c+James+J+Peters+VA+Hospital)

- - Robert Striker, M.D., Ph.D.

William S. Middleton Memorial Veterans Hospital, 2500 Overlook Terrace, Madison, WI 53705

- GWAS & Downstream Analysis
  - Jennifer Huffman, Ph.D.

VA Boston Healthcare System, 150 S. Huntington Avenue, Boston, MA 02130

- - Yan Sun, Ph.D.

Atlanta VA Medical Center, 1670 Clairmont Road, Decatur, GA 30033

- Pharmacogenomics
  - Adriana Hung, M.D., M.P.H.

VA Tennessee Valley Healthcare System, 1310 24th Avenue, South Nashville, TN 37212

- - Sony Tuteja, Pharm.D., M.S.

Philadelphia VA Medical Center, 3900 Woodland Avenue, Philadelphia, PA 19104

- VA COVID-19 Shared Data Resource – Scott L. DuVall, Ph.D.; Kristine E. Lynch, Ph.D.; Elise Gatsby, M.P.H.

VA Informatics and Computing Infrastructure (VINCI), VA Salt Lake City Health Care System, 500 Foothill Drive, Salt Lake City, UT 84148

- MVP COVID-19 Data Core – Kelly Cho, M.P.H., Ph.D.; Lauren Costa, M.P.H.; Anne Yuk-Lam Ho, M.P.H.; Rebecca Song, M.P.H.

VA Boston Healthcare System, 150 S. Huntington Avenue, Boston, MA 02130

**VA Million Veteran Program**

**MVP Executive Committee**

- Co-Chair: J. Michael Gaziano, M.D., M.P.H.

VA Boston Healthcare System, 150 S. Huntington Avenue, Boston, MA 02130

- Co-Chair: Sumitra Muralidhar, Ph.D.

US Department of Veterans Affairs, 810 Vermont Avenue NW, Washington, DC 20420

- Rachel Ramoni, D.M.D., Sc.D., Chief VA Research and Development Officer

US Department of Veterans Affairs, 810 Vermont Avenue NW, Washington, DC 20420

- Jean Beckham, Ph.D.

Durham VA Medical Center, 508 Fulton Street, Durham, NC 27705

- Kyong-Mi Chang, M.D.

Philadelphia VA Medical Center, 3900 Woodland Avenue, Philadelphia, PA 19104

- Christopher J. O’Donnell, M.D., M.P.H.

VA Boston Healthcare System, 150 S. Huntington Avenue, Boston, MA 02130

- Philip S. Tsao, Ph.D.

VA Palo Alto Health Care System, 3801 Miranda Avenue, Palo Alto, CA 94304

- James Breeling, M.D., Ex-Officio

US Department of Veterans Affairs, 810 Vermont Avenue NW, Washington, DC 20420

- Grant Huang, Ph.D., Ex-Officio

US Department of Veterans Affairs, 810 Vermont Avenue NW, Washington, DC 20420

- Juan P. Casas, M.D., Ph.D., Ex-Officio

VA Boston Healthcare System, 150 S. Huntington Avenue, Boston, MA 02130

**MVP Program Office**

- Sumitra Muralidhar, Ph.D.

US Department of Veterans Affairs, 810 Vermont Avenue NW, Washington, DC 20420

- Jennifer Moser, Ph.D.

US Department of Veterans Affairs, 810 Vermont Avenue NW, Washington, DC 20420

**MVP Recruitment/Enrollment**

- Recruitment/Enrollment Director/Deputy Director, Boston – Stacey B. Whitbourne, Ph.D.; Jessica V. Brewer, M.P.H.

VA Boston Healthcare System, 150 S. Huntington Avenue, Boston, MA 02130

- MVP Coordinating Centers
  - Clinical Epidemiology Research Center (CERC), West Haven – Mihaela Aslan, Ph.D.

West Haven VA Medical Center, 950 Campbell Avenue, West Haven, CT 06516

- - Cooperative Studies Program Clinical Research Pharmacy Coordinating Center, Albuquerque – Todd Connor, Pharm.D.; Dean P. Argyres, B.S., M.S.

New Mexico VA Health Care System, 1501 San Pedro Drive SE, Albuquerque, NM 87108

- - Genomics Coordinating Center, Palo Alto – Philip S. Tsao, Ph.D.

VA Palo Alto Health Care System, 3801 Miranda Avenue, Palo Alto, CA 94304

- - MVP Boston Coordinating Center, Boston - J. Michael Gaziano, M.D., M.P.H.

VA Boston Healthcare System, 150 S. Huntington Avenue, Boston, MA 02130

- - MVP Information Center, Canandaigua – Brady Stephens, M.S.

Canandaigua VA Medical Center, 400 Fort Hill Avenue, Canandaigua, NY 14424

- VA Central Biorepository, Boston – Mary T. Brophy M.D., M.P.H.; Donald E. Humphries, Ph.D.; Luis E. Selva, Ph.D.

VA Boston Healthcare System, 150 S. Huntington Avenue, Boston, MA 02130

- MVP Informatics, Boston – Nhan Do, M.D.; Shahpoor (Alex) Shayan, M.S.

VA Boston Healthcare System, 150 S. Huntington Avenue, Boston, MA 02130

- MVP Data Operations/Analytics, Boston – Kelly Cho, M.P.H., Ph.D.

VA Boston Healthcare System, 150 S. Huntington Avenue, Boston, MA 02130

- Director of Regulatory Affairs – Lori Churby, B.S.

VA Palo Alto Health Care System, 3801 Miranda Avenue, Palo Alto, CA 94304

**MVP Science**

- Science Operations – Christopher J. O’Donnell, M.D., M.P.H.

VA Boston Healthcare System, 150 S. Huntington Avenue, Boston, MA 02130

- Genomics Core - Christopher J. O’Donnell, M.D., M.P.H.

VA Boston Healthcare System, 150 S. Huntington Avenue, Boston, MA 02130

Saiju Pyarajan Ph.D.

VA Boston Healthcare System, 150 S. Huntington Avenue, Boston, MA 02130

Philip S. Tsao, Ph.D.

VA Palo Alto Health Care System, 3801 Miranda Avenue, Palo Alto, CA 94304

- Data Core - Kelly Cho, M.P.H, Ph.D.

VA Boston Healthcare System, 150 S. Huntington Avenue, Boston, MA 02130

- VA Informatics and Computing Infrastructure (VINCI) – Scott L. DuVall, Ph.D.

VA Salt Lake City Health Care System, 500 Foothill Drive, Salt Lake City, UT 84148

- Data and Computational Sciences – Saiju Pyarajan, Ph.D.

VA Boston Healthcare System, 150 S. Huntington Avenue, Boston, MA 02130

- Statistical Genetics – Elizabeth Hauser, Ph.D.

Durham VA Medical Center, 508 Fulton Street, Durham, NC 27705

Yan Sun, Ph.D.

Atlanta VA Medical Center, 1670 Clairmont Road, Decatur, GA 30033

Hongyu Zhao, Ph.D.

West Haven VA Medical Center, 950 Campbell Avenue, West Haven, CT 06516

**Current MVP Local Site Investigators**

- Atlanta VA Medical Center (Peter Wilson, M.D.)

1670 Clairmont Road, Decatur, GA 30033

- Bay Pines VA Healthcare System (Rachel McArdle, Ph.D.)

10,000 Bay Pines Blvd Bay Pines, FL 33744

- Birmingham VA Medical Center (Louis Dellitalia, M.D.)

700 S. 19th Street, Birmingham AL 35233

- Central Western Massachusetts Healthcare System (Kristin Mattocks, Ph.D., M.P.H.)

421 North Main Street, Leeds, MA 01053

- Cincinnati VA Medical Center (John Harley, M.D., Ph.D.)

3200 Vine Street, Cincinnati, OH 45220

- Clement J. Zablocki VA Medical Center (Jeffrey Whittle, M.D., M.P.H.)

5000 West National Avenue, Milwaukee, WI 53295

- VA Northeast Ohio Healthcare System (Frank Jacono, M.D.)

10701 East Boulevard, Cleveland, OH 44106

- Durham VA Medical Center (Jean Beckham, Ph.D.)

508 Fulton Street, Durham, NC 27705

- Edith Nourse Rogers Memorial Veterans Hospital (John Wells., Ph.D.)

200 Springs Road, Bedford, MA 01730

- Edward Hines, Jr. VA Medical Center (Salvador Gutierrez, M.D.)

5000 South 5th Avenue, Hines, IL 60141

- Veterans Health Care System of the Ozarks (Gretchen Gibson, D.D.S., M.P.H.)

1100 North College Avenue, Fayetteville, AR 72703

- Fargo VA Health Care System (Kimberly Hammer, Ph.D.)

2101 N. Elm, Fargo, ND 58102

- VA Health Care Upstate New York (Laurence Kaminsky, Ph.D.)

113 Holland Avenue, Albany, NY 12208

- New Mexico VA Health Care System (Gerardo Villareal, M.D.)

1501 San Pedro Drive, S.E. Albuquerque, NM 87108

- VA Boston Healthcare System (Scott Kinlay, M.B.B.S., Ph.D.)

150 S. Huntington Avenue, Boston, MA 02130

- VA Western New York Healthcare System (Junzhe Xu, M.D.)

3495 Bailey Avenue, Buffalo, NY 14215-1199

- Ralph H. Johnson VA Medical Center (Mark Hamner, M.D.)

109 Bee Street, Mental Health Research, Charleston, SC 29401

- Columbia VA Health Care System (Roy Mathew, M.D.)

6439 Garners Ferry Road, Columbia, SC 29209

- VA North Texas Health Care System (Sujata Bhushan, M.D.)

4500 S. Lancaster Road, Dallas, TX 75216

- Hampton VA Medical Center (Pran Iruvanti, D.O., Ph.D.)

100 Emancipation Drive, Hampton, VA 23667

- Richmond VA Medical Center (Michael Godschalk, M.D.)

1201 Broad Rock Blvd., Richmond, VA 23249

- Iowa City VA Health Care System (Zuhair Ballas, M.D.)

601 Highway 6 West, Iowa City, IA 52246-2208

- Eastern Oklahoma VA Health Care System (Douglas Ivins, M.D.)

1011 Honor Heights Drive, Muskogee, OK 74401

- James A. Haley Veterans’ Hospital (Stephen Mastorides, M.D.)

13000 Bruce B. Downs Blvd, Tampa, FL 33612

- James H. Quillen VA Medical Center (Jonathan Moorman, M.D., Ph.D.)

Corner of Lamont & Veterans Way, Mountain Home, TN 37684

- John D. Dingell VA Medical Center (Saib Gappy, M.D.)

4646 John R Street, Detroit, MI 48201

- Louisville VA Medical Center (Jon Klein, M.D., Ph.D.)

800 Zorn Avenue, Louisville, KY 40206

- Manchester VA Medical Center (Nora Ratcliffe, M.D.)

718 Smyth Road, Manchester, NH 03104

- Miami VA Health Care System (Hermes Florez, M.D., Ph.D.)

1201 NW 16th Street, 11 GRC, Miami FL 33125

- Michael E. DeBakey VA Medical Center (Olaoluwa Okusaga, M.D.)

2002 Holcombe Blvd, Houston, TX 77030

- Minneapolis VA Health Care System (Maureen Murdoch, M.D., M.P.H.)

One Veterans Drive, Minneapolis, MN 55417

- N. FL/S. GA Veterans Health System (Peruvemba Sriram, M.D.)

1601 SW Archer Road, Gainesville, FL 32608

- Northport VA Medical Center (Shing Shing Yeh, Ph.D., M.D.)

79 Middleville Road, Northport, NY 11768

- Overton Brooks VA Medical Center (Neeraj Tandon, M.D.)

510 East Stoner Ave, Shreveport, LA 71101

- Philadelphia VA Medical Center (Darshana Jhala, M.D.)

3900 Woodland Avenue, Philadelphia, PA 19104

- Phoenix VA Health Care System (Samuel Aguayo, M.D.)

650 E. Indian School Road, Phoenix, AZ 85012

- Portland VA Medical Center (David Cohen, M.D.)

3710 SW U.S. Veterans Hospital Road, Portland, OR 97239

- Providence VA Medical Center (Satish Sharma, M.D.)

830 Chalkstone Avenue, Providence, RI 02908

- Richard Roudebush VA Medical Center (Suthat Liangpunsakul, M.D., M.P.H.)

1481 West 10th Street, Indianapolis, IN 46202

- Salem VA Medical Center (Kris Ann Oursler, M.D.)

1970 Roanoke Blvd, Salem, VA 24153

- San Francisco VA Health Care System (Mary Whooley, M.D.)

4150 Clement Street, San Francisco, CA 94121

- South Texas Veterans Health Care System (Sunil Ahuja, M.D.)

7400 Merton Minter Boulevard, San Antonio, TX 78229

- Southeast Louisiana Veterans Health Care System (Joseph Constans, Ph.D.)

2400 Canal Street, New Orleans, LA 70119

- Southern Arizona VA Health Care System (Paul Meyer, M.D., Ph.D.)

3601 S 6th Avenue, Tucson, AZ 85723

- Sioux Falls VA Health Care System (Jennifer Greco, M.D.)

2501 W 22nd Street, Sioux Falls, SD 57105

- St. Louis VA Health Care System (Michael Rauchman, M.D.)

915 North Grand Blvd, St. Louis, MO 63106

- Syracuse VA Medical Center (Richard Servatius, Ph.D.)

800 Irving Avenue, Syracuse, NY 13210

- VA Eastern Kansas Health Care System (Melinda Gaddy, Ph.D.)

4101 S 4th Street Trafficway, Leavenworth, KS 66048

- VA Greater Los Angeles Health Care System (Agnes Wallbom, M.D., M.S.)

11301 Wilshire Blvd, Los Angeles, CA 90073

- VA Long Beach Healthcare System (Timothy Morgan, M.D.)

5901 East 7th Street Long Beach, CA 90822

- VA Maine Healthcare System (Todd Stapley, D.O.)

1 VA Center, Augusta, ME 04330

- VA New York Harbor Healthcare System (Scott Sherman, M.D., M.P.H.)

423 East 23rd Street, New York, NY 10010

- VA Pacific Islands Health Care System (George Ross, M.D.)

459 Patterson Rd, Honolulu, HI 96819

- VA Palo Alto Health Care System (Philip Tsao, Ph.D.)

3801 Miranda Avenue, Palo Alto, CA 94304-1290

- VA Pittsburgh Health Care System (Patrick Strollo, Jr., M.D.)

University Drive, Pittsburgh, PA 15240

- VA Puget Sound Health Care System (Edward Boyko, M.D.)

1660 S. Columbian Way, Seattle, WA 98108-1597

- VA Salt Lake City Health Care System (Laurence Meyer, M.D., Ph.D.)

500 Foothill Drive, Salt Lake City, UT 84148

- VA San Diego Healthcare System (Samir Gupta, M.D., M.S.C.S.)

3350 La Jolla Village Drive, San Diego, CA 92161

- VA Sierra Nevada Health Care System (Mostaqul Huq, Pharm.D., Ph.D.)

975 Kirman Avenue, Reno, NV 89502

- VA Southern Nevada Healthcare System (Joseph Fayad, M.D.)

6900 North Pecos Road, North Las Vegas, NV 89086

- VA Tennessee Valley Healthcare System (Adriana Hung, M.D., M.P.H.)

1310 24th Avenue, South Nashville, TN 37212

- Washington DC VA Medical Center (Jack Lichy, M.D., Ph.D.)

50 Irving St, Washington, D. C. 20422

- W.G. (Bill) Hefner VA Medical Center (Robin Hurley, M.D.)

1601 Brenner Ave, Salisbury, NC 28144

- White River Junction VA Medical Center (Brooks Robey, M.D.)

163 Veterans Drive, White River Junction, VT 05009

- William S. Middleton Memorial Veterans Hospital (Robert Striker, M.D., Ph.D.)

2500 Overlook Terrace, Madison, WI 53705

**Supplemental Methods**

Study participants

The VA Million Veteran Program (MVP) is an ongoing longitudinal study that began in 2011 and was designed to study genetic and non-genetic determinants of health diseases among U.S. Veterans recruited from 63 Veterans Health Administration (VA) medical facilities (Gaziano et al., 2016). Veterans aged 18 years and older are recruited into MVP where participants are linked to VA electronic health records (EHR), complete a questionnaire, and submit a blood sample at enrollment. We included MVP participants who had genome-wide genotype and EHR-extracted COVID-19 related phenotype data available and who were alive as of February 29, 2020.

Demographic and clinical characteristics were obtained from the VA EHR housed within the VA’s Corporate Data Warehouse (CDW) and the MVP central data repository, curated EHR and survey data available only for MVP research studies. Age and sex for participants were obtained from the MVP Baseline Survey and supplemented with patient health records from CDW when self-reported demographics were not available.

MVP received ethical/study protocol approval by the VA Central Institutional Review Board and informed consent was obtained for all participants.

Genetic data, quality control, and imputation

Study participants were genotyped using a customized Affymetrix Axiom biobank array (the MVP 1.0 Genotyping Array), containing 723,305 variants (Klarin et al., 2018; Hunter-Zinck et al., 2020). Imputation was performed to a hybrid imputation panel comprised of the African Genome Resources panel (https://imputation.sanger.ac.uk/?about=1#referencepanels) and 1000G v3p5.

Population-specific principal components (PCs) were computed using EIGENSOFT v.6 (Price et al., 2006). The harmonized race/ethnicity and genetic ancestry (HARE) approach, developed by MVP, was used to assign individuals to populations or groups. This machine learning algorithm leverages information from both the self-identified race/ethnicity data from the survey and data from the genome-wide array to create respective variables for downstream analyses (Fang et al., 2019). Based on HARE, we categorized Veterans into three mutually exclusive groups: (1) non-Hispanic White (White), (2) non-Hispanic Black (Black), and (3) Hispanic or Latino (Hispanic). Kinship was inferred to identify cryptic relatedness using KING v.2.0 (Manichaikul et al., 2010). For each pair of first and second-degree relatives identified (kinship coefficient ≥ 0.0884), one individual was excluded while preferentially retaining those who tested positive for SARS-CoV-2.

COVID-19 definitions

Cases of COVID-19 among MVP participants were identified using an algorithm developed by the VA COVID National Surveillance Tool (NST) (Chapman et al., 2020). The NST classified COVID-19 cases as positive (+) or negative (-) based on reverse transcription polymerase chain reaction (rRT-PCR) laboratory test results conducted at VA clinics, supplemented with Natural Language Processing (NLP) on clinical documents. The algorithm to identify COVID-19 patients is continually updated to ensure new annotations of COVID-19 are captured from the clinical notes, with chart reviews performed periodically to validate the algorithm. We used the data available in February 2021. COVID-19-related hospitalizations were defined as admissions from 7 days before up to 30 days after a patient's first positive test for SARS-CoV-2 test. Among the 631,019 MVP participants with release 4 imputed data in one of the three mutually exclusive HARE groups, we excluded participants who died before March 1, 2020 (n=96,807) and one participant from each pair of related individuals (n=21,176). Among the remaining 513,036 participants, 19,168 tested positive for SARS-CoV-2 between March 1, 2020 and February 2, 2021.

ABO blood type

ABO blood type calling was inferred using 4 genetic variants, rs8176746, rs507666, rs687289, and rs8176719 (Pare et al., 2008; Severe Covid GWAS Group et al., 2020) in all MVP participants with genotyping information. To evaluate the accuracy of ABO blood type inferred by genotype, we calculated the concordance between our genotype inferred ABO blood groups and the serology based determination of ABO blood group for a subset of 532 genotyped MVP participants who underwent ABO typing using serologic tests in the context of clinical care.

Statistical Analysis

We performed single variant association, separately by HARE-assigned group, for genotyped and imputed variants using four outcomes: 1) COVID-19 positivity as defined by positive COVID-19 test compared with all other MVP participants (POS vs POP), 2) Individuals who were hospitalized for COVID-19 compared with all other MVP participants, which includes individuals that tested positive for COVID-19 but were not hospitalized (HOS vs POP), 3) Individuals who were hospitalized for COVID-19 compared with individuals who tested positive for COVID-19 but were not hospitalized (HOS vs NOT), and 4) Individuals who were hospitalized for COVID-19 with high-flow oxygen or died of COVID-19 (severe COVID-19) compared with all other MVP participants (SEV vs POP). Participants with missing imputed genotype data, age, or sex were excluded from analyses.

We excluded variants with population-specific minor allele frequency < 0.5% and imputation quality < 0.3 before performing single variant association analyses within each of the HARE-assigned groups. We associated each genetic variant with COVID-19 positivity and hospitalization outcomes using logistic regression in PLINK v2 (Chang et al., 2015). All analyses were adjusted for age, age^2^, sex, age*sex, and 15 population-specific PCs. Fixed-effects meta-analysis was performed across HARE-assigned groups using GWAMA (Magi and Morris, 2010). Genomic control was applied to each population prior to meta-analysis but was not applied again to the meta-analysis results themselves. Population-specific results presented are prior to genomic control.

We obtained COVID-19 Host Genetics Initiative (HGI) summary statistics (Release 5) for the multi-population and the White-only meta-analyses, excluding MVP and 23&Me data, for replication. We applied a Multi-marker Analysis of GenoMic Annotation (MAGMA) v1.09 for gene-based analysis, as implemented in FUMA (de Leeuw et al., 2015, 2018; Watanabe et al., 2017) using the 1000Genomes Phase 3 European reference panel and a window size of 10kb +/- the gene start and end. MAGMA gene-set analyses were run on 10,678 gene sets (curated gene sets: 4,761, GO terms: 5,917) from MsigDB v6.2.

We studied the association between ABO blood type and COVID-19 using logistic regression adjusted for age and sex. We tested four COVID outcomes: 1) POS vs POP, 2) HOS vs POP, 3) HOS vs NOT, and 4) SEV vs POP. We performed the analysis for each HARE-assigned group as well as for all groups combined.

**Supplemental Figures**

**Figure S1 - QQ plots of genome-wide analyses**

*COVID-19 Positivity*

COVID-19 POS vs POP in META COVID-19 POS vs POP in WHITE


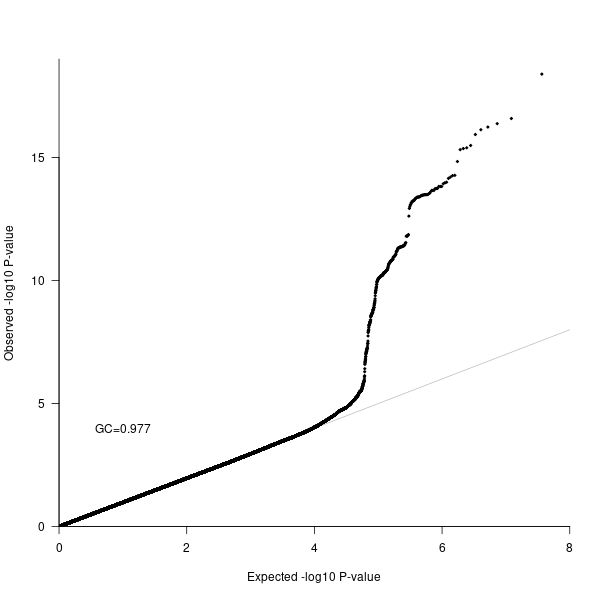

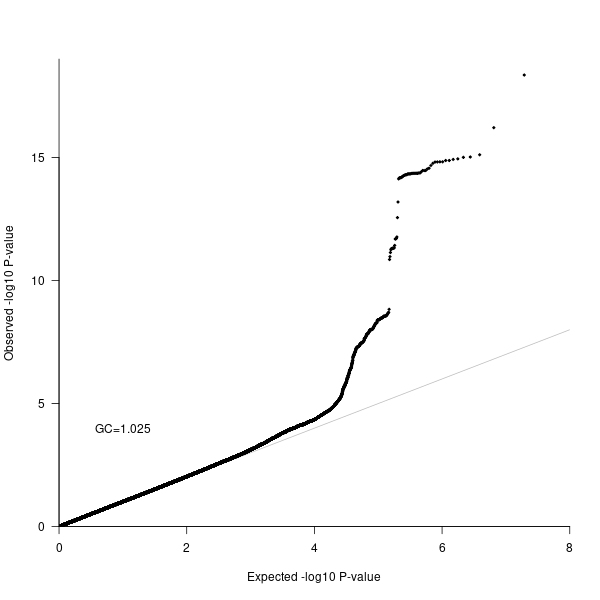


COVID-19 POS vs POP in BLACK COVID-19 POS vs POP in HISPANIC


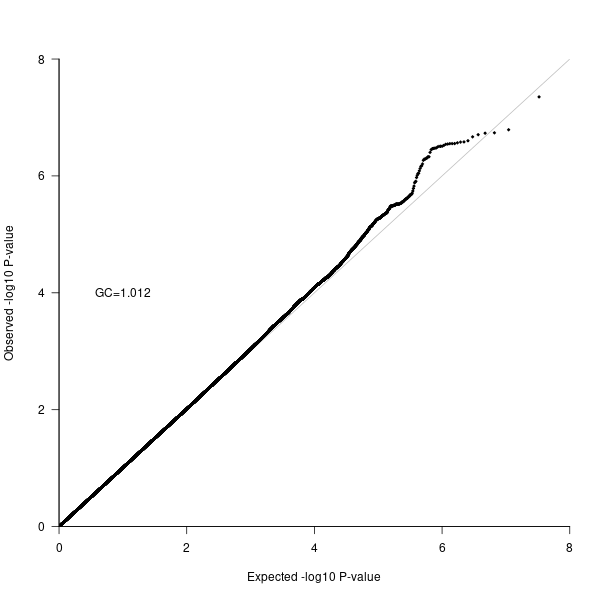

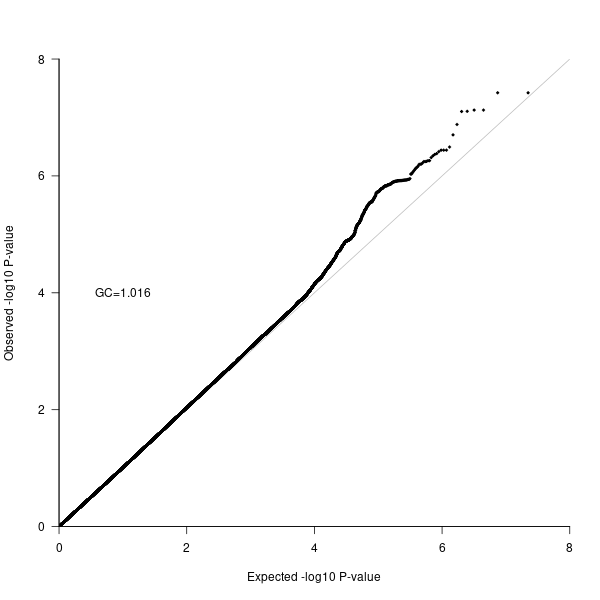


*COVID-19 Hospitalize vs population controls*

COVID-19 HOS vs POP in META COVID-19 HOS vs POP in WHITE


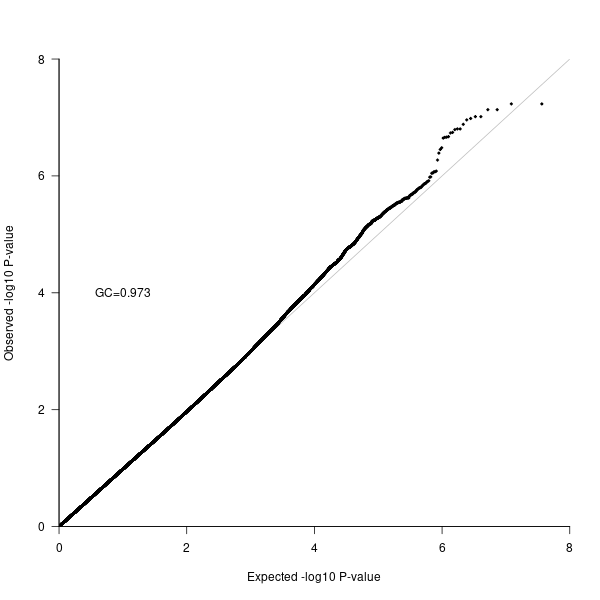

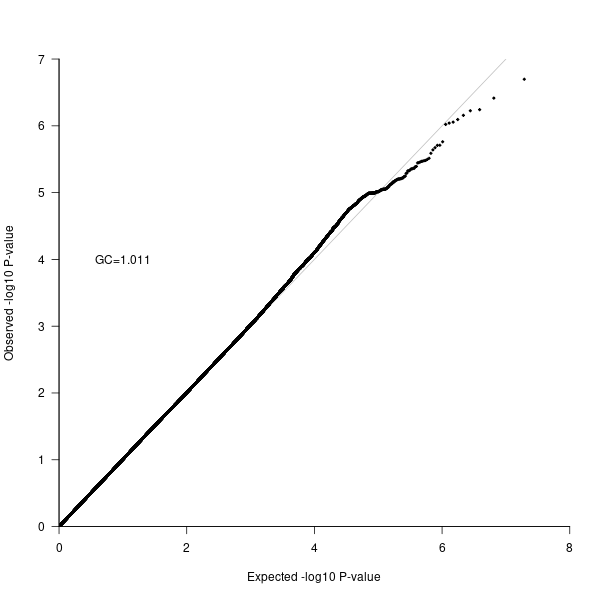


COVID-19 HOS vs POP in BLACK COVID-19 HOS vs POP in HISPANIC


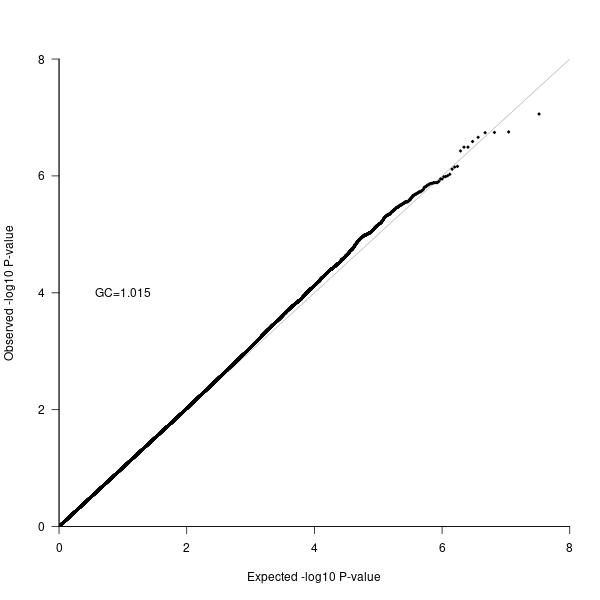

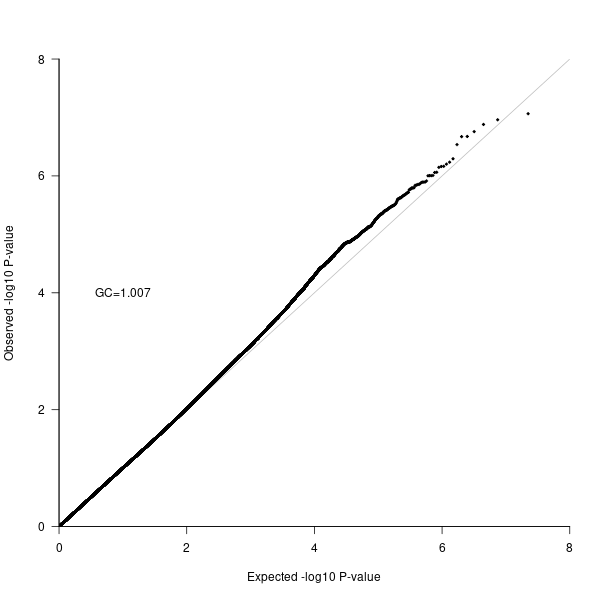


*Hospitalize vs not hospitalized COVID-19 patients*

COVID-19 HOS vs NOT in META COVID-19 HOS vs NOT in WHITE


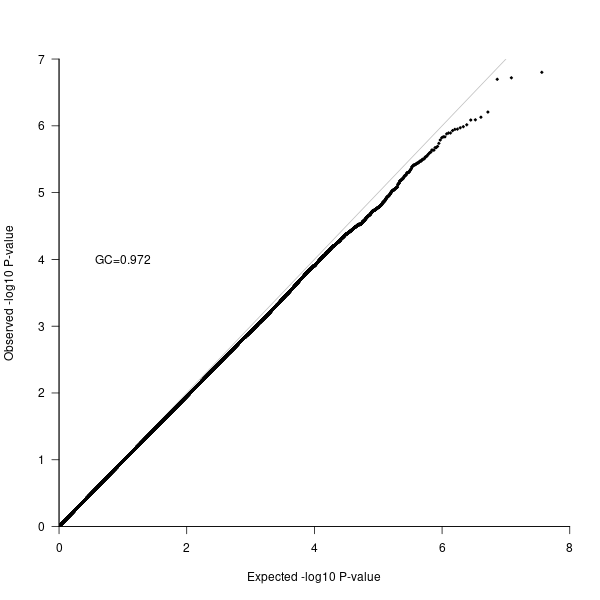

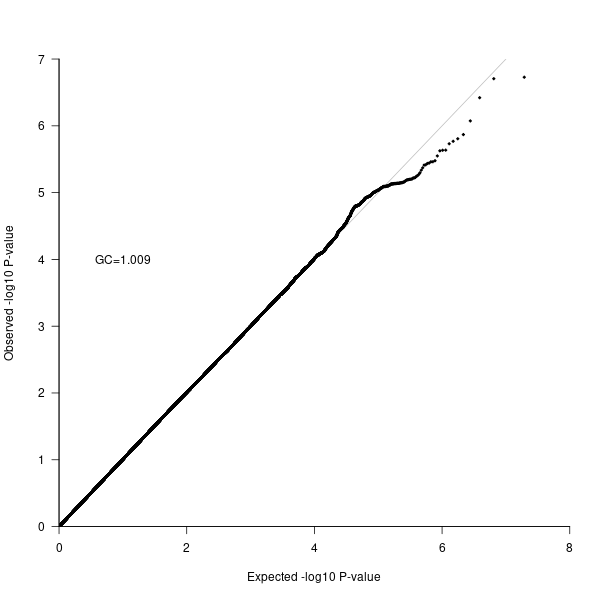


COVID-19 HOS vs NOT in BLACK COVID-19 HOS vs NOT in HISPANIC


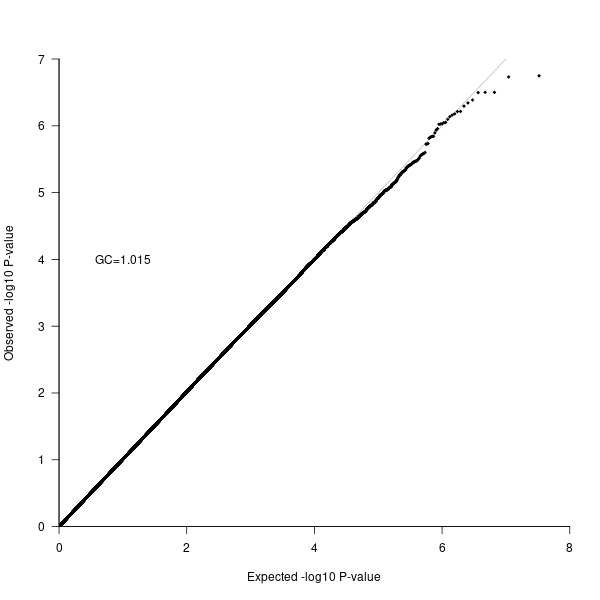

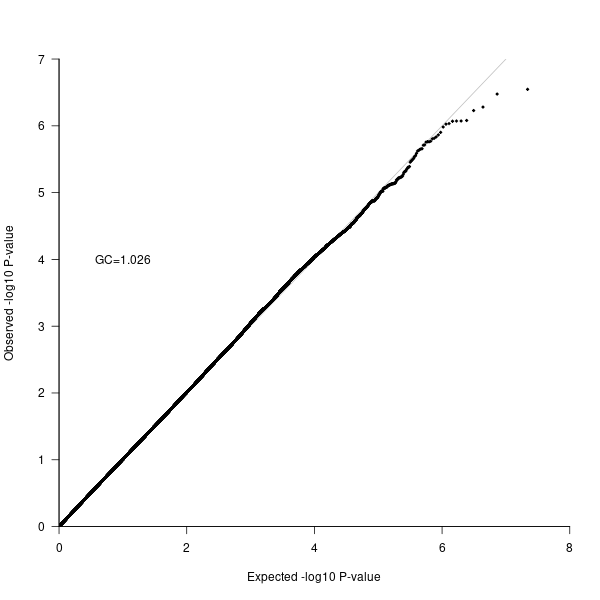


*Severe COVID-19 (Hospitalization with respiratory support or death)*

COVID-19 SEV vs POP in META COVID-19 SEV vs POP in WHITE


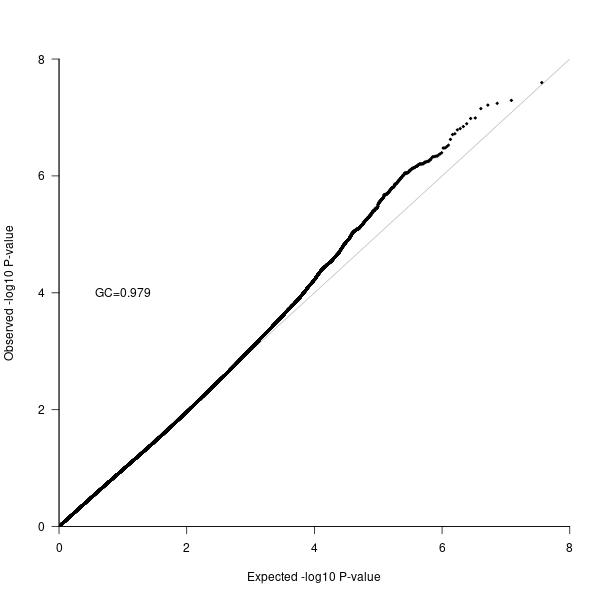
 **
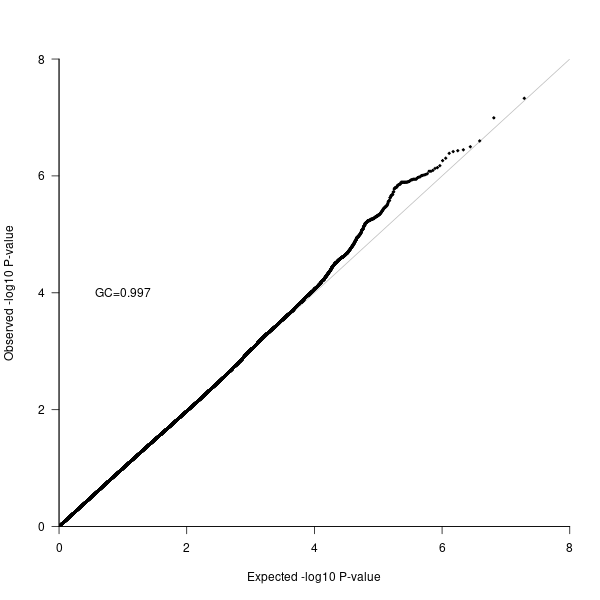
**

COVID-19 SEV vs POP in BLACK COVID-19 SEV vs POP in HISPANICS

**
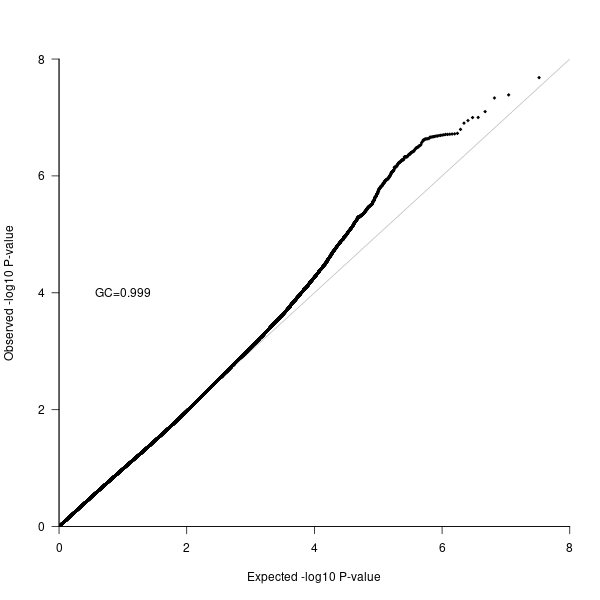
** **
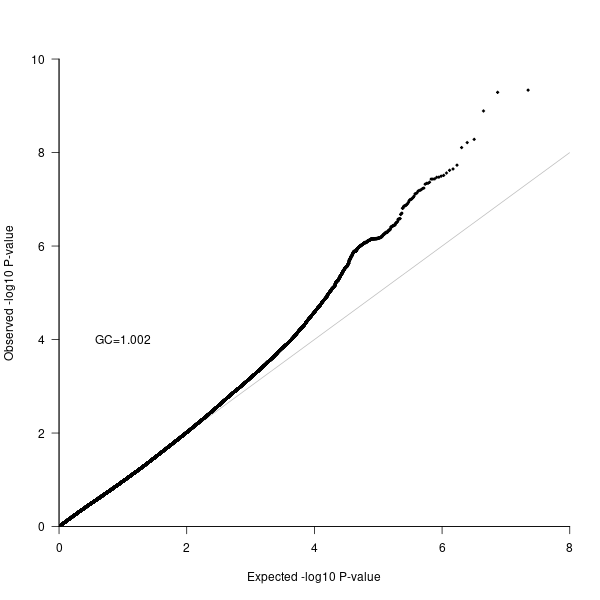
**

**Figure S2 – Manhattan plots of genome-wide analyses**

POS vs POP in META


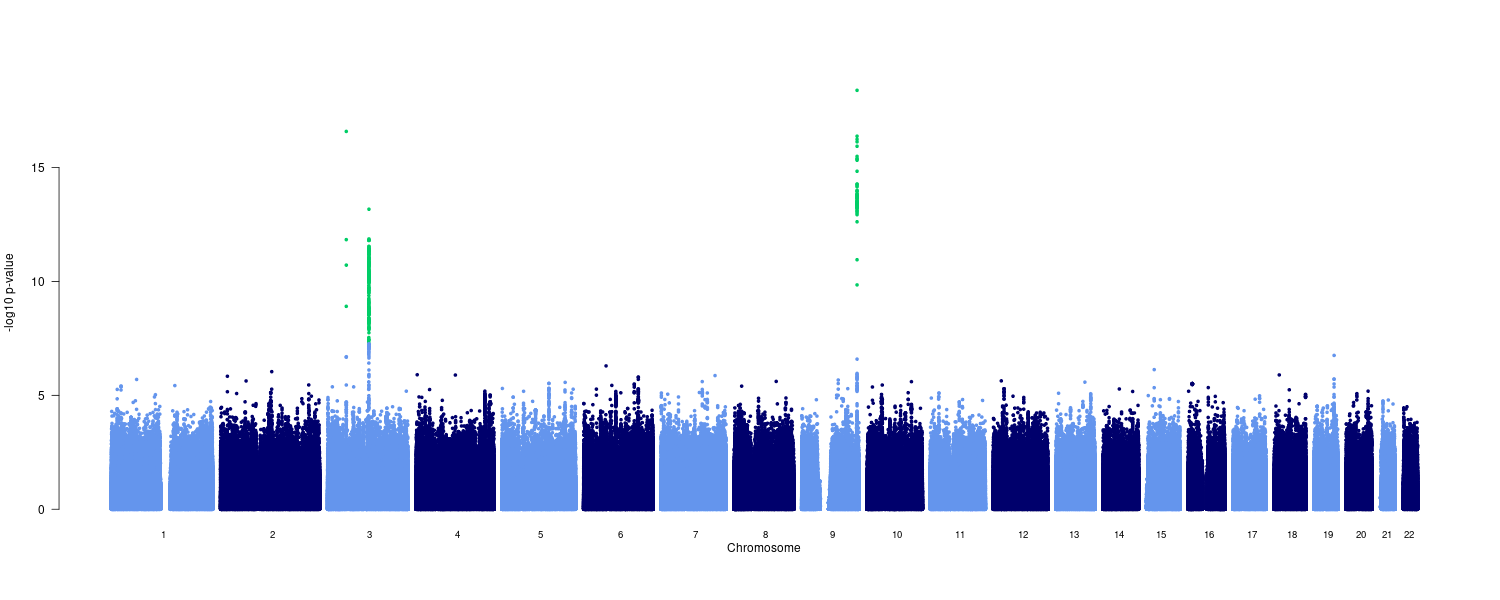


POS vs POP in WHITE
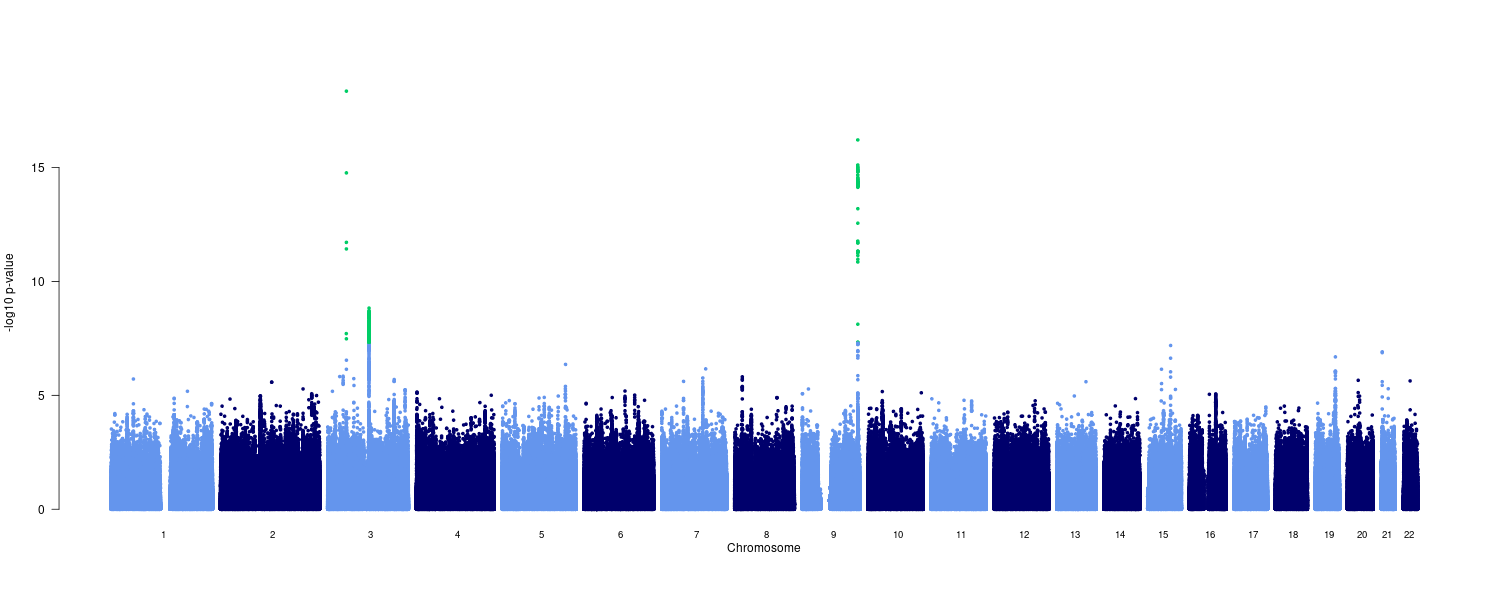


POS vs POP in BLACK


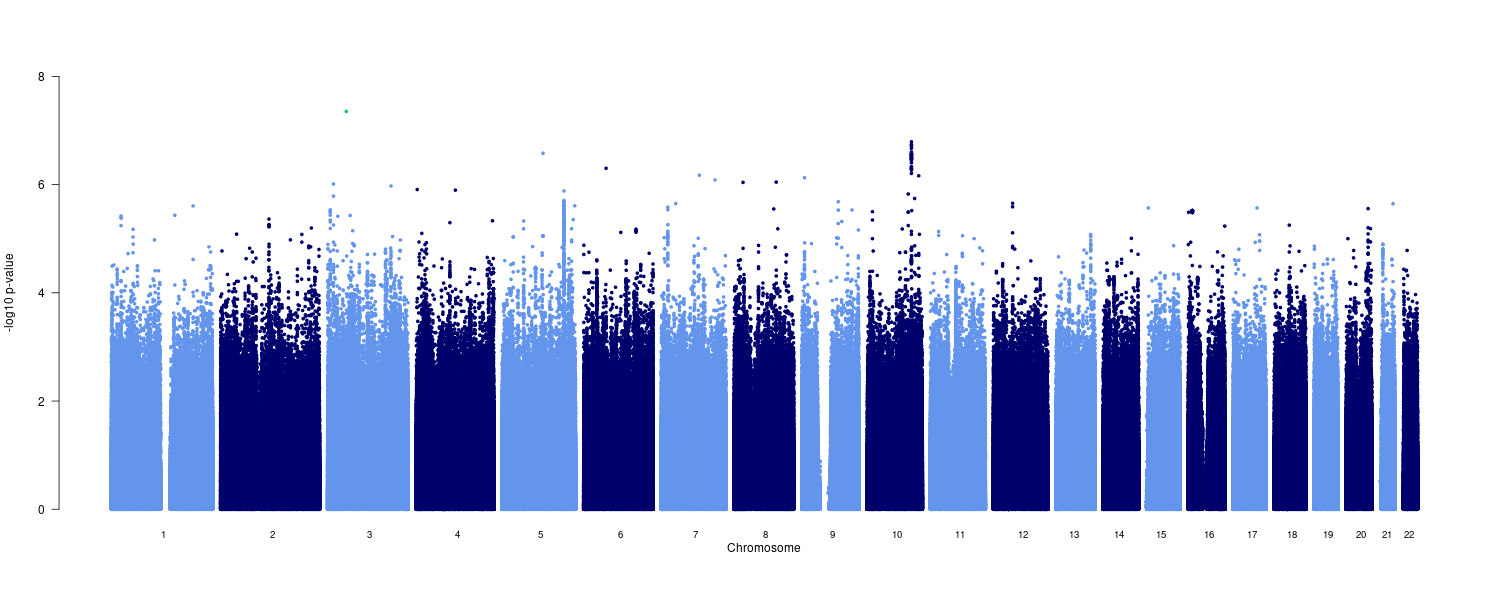


POS vs POP in HISPANIC


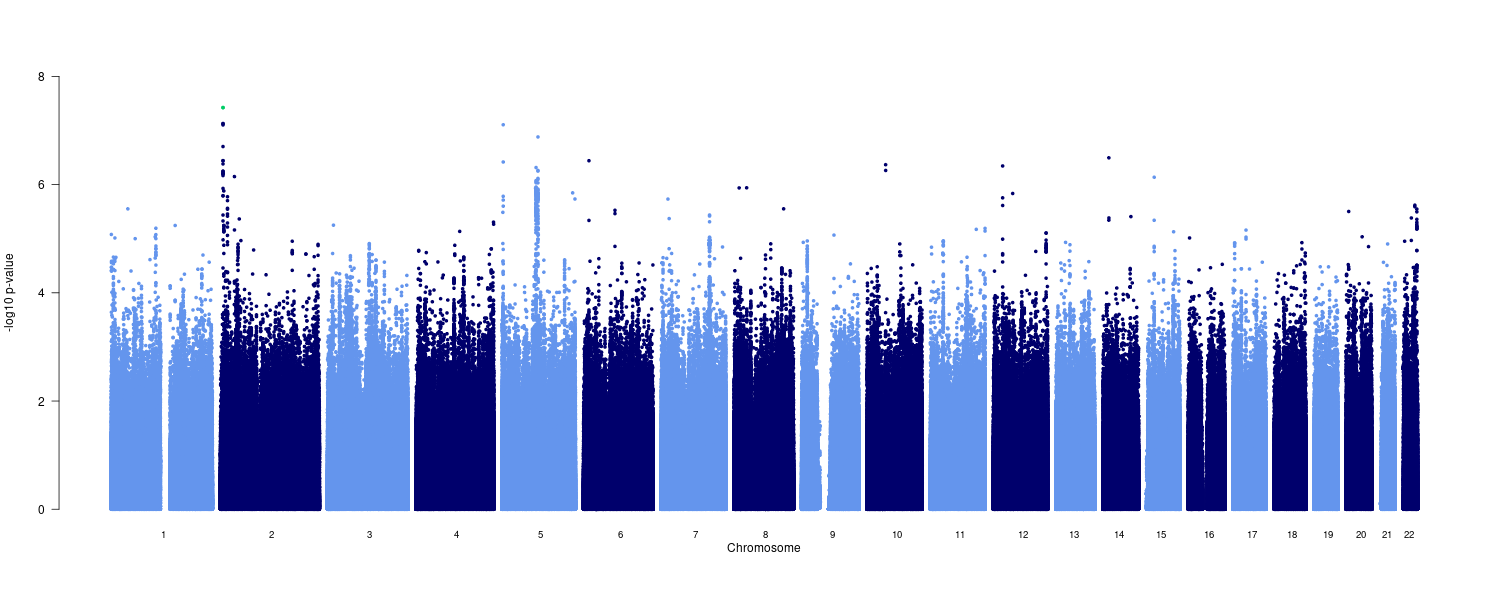


HOS vs POP in META


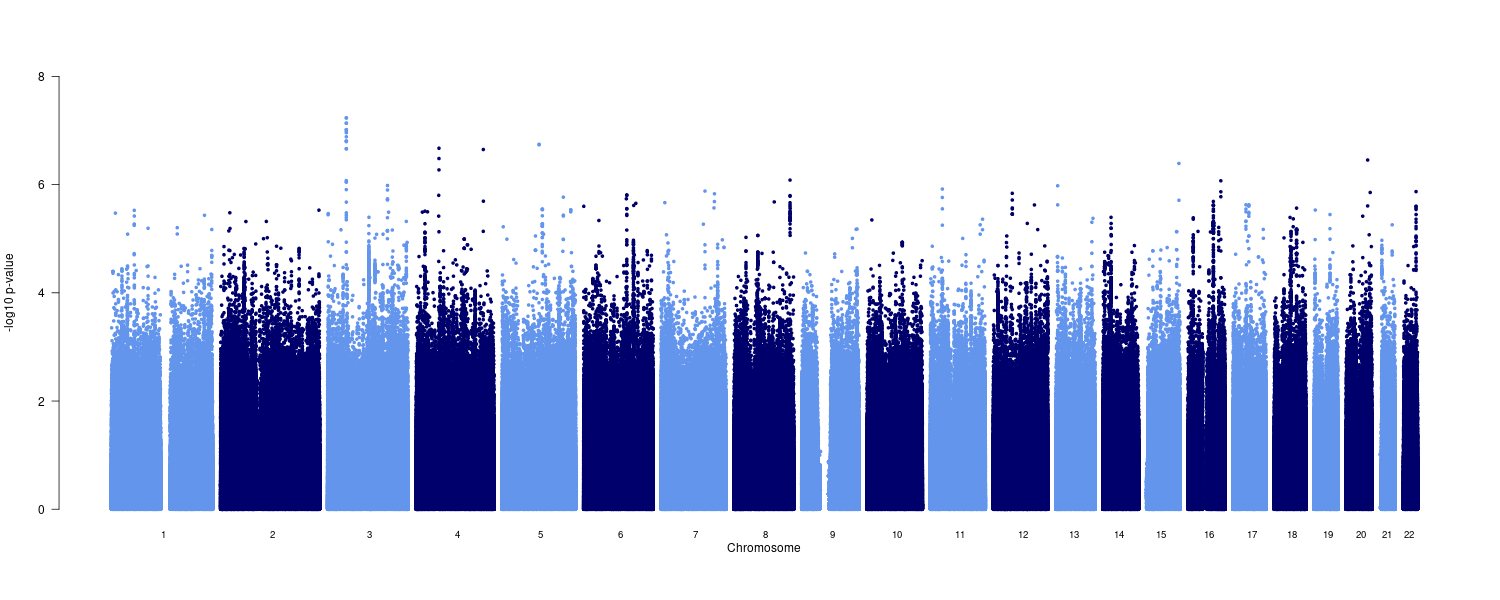


HOS vs POP in WHITE


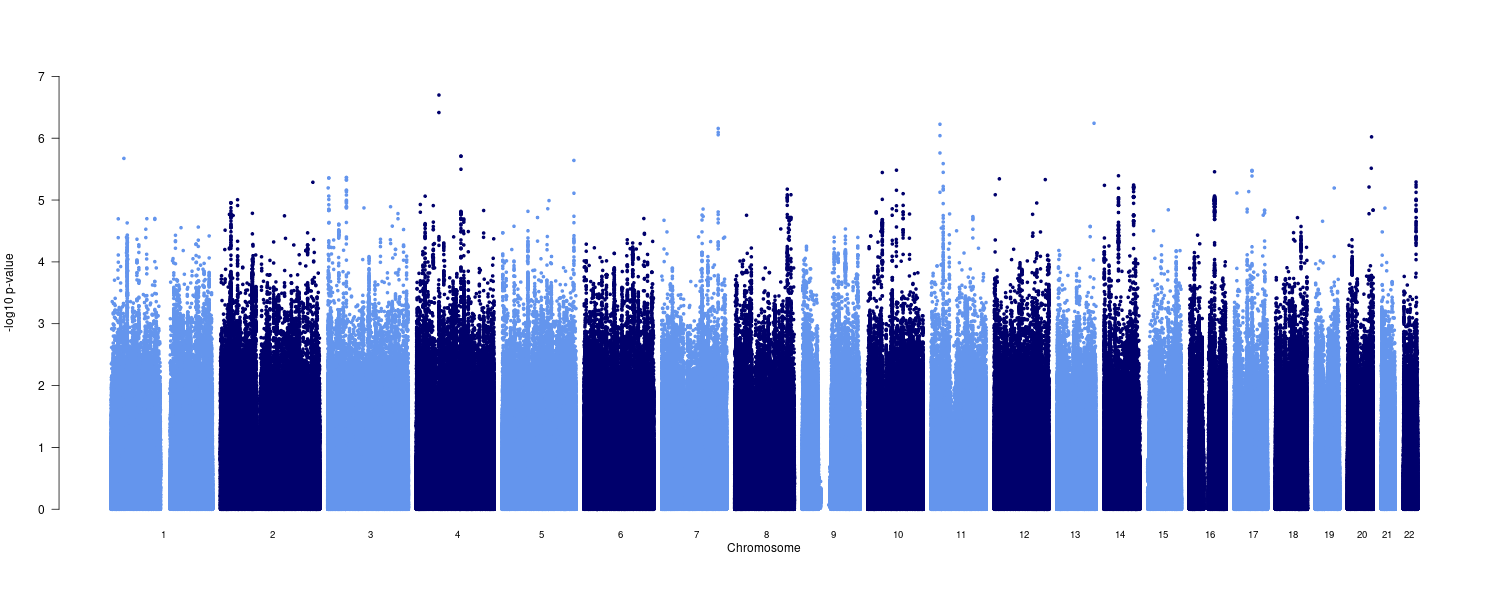


HOS vs POP in BLACK


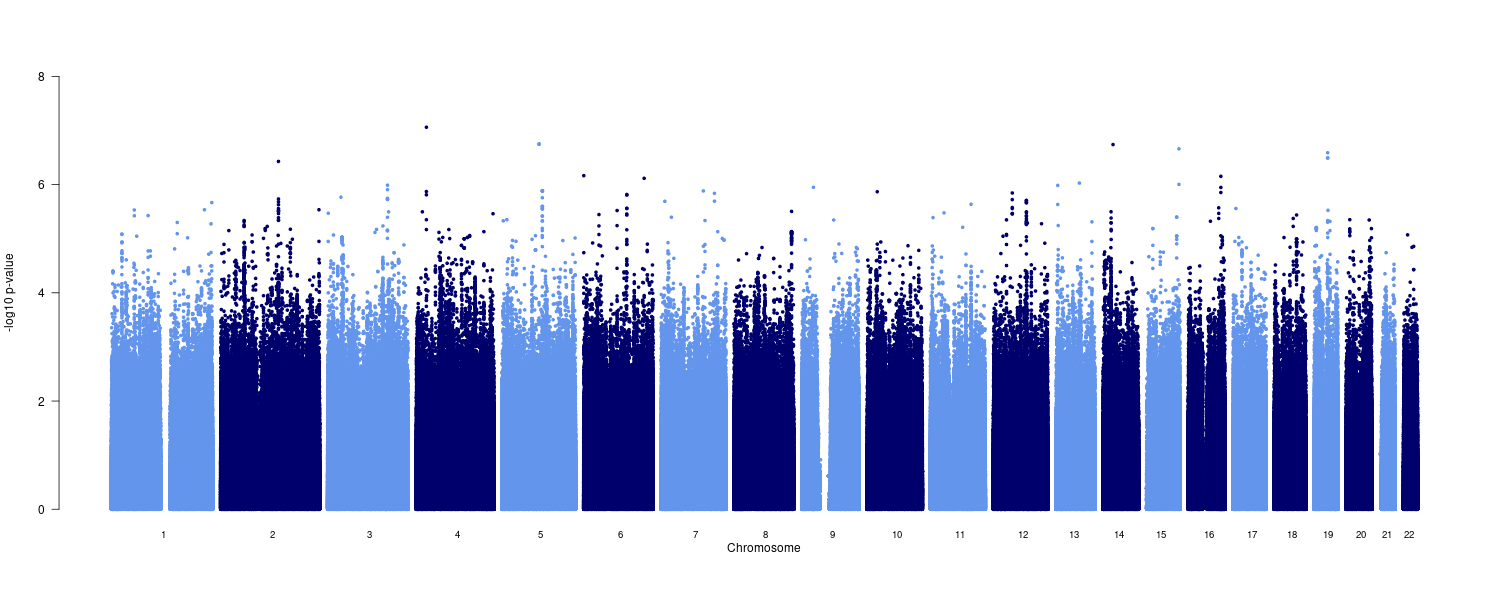


HOS vs POP in HISPANIC


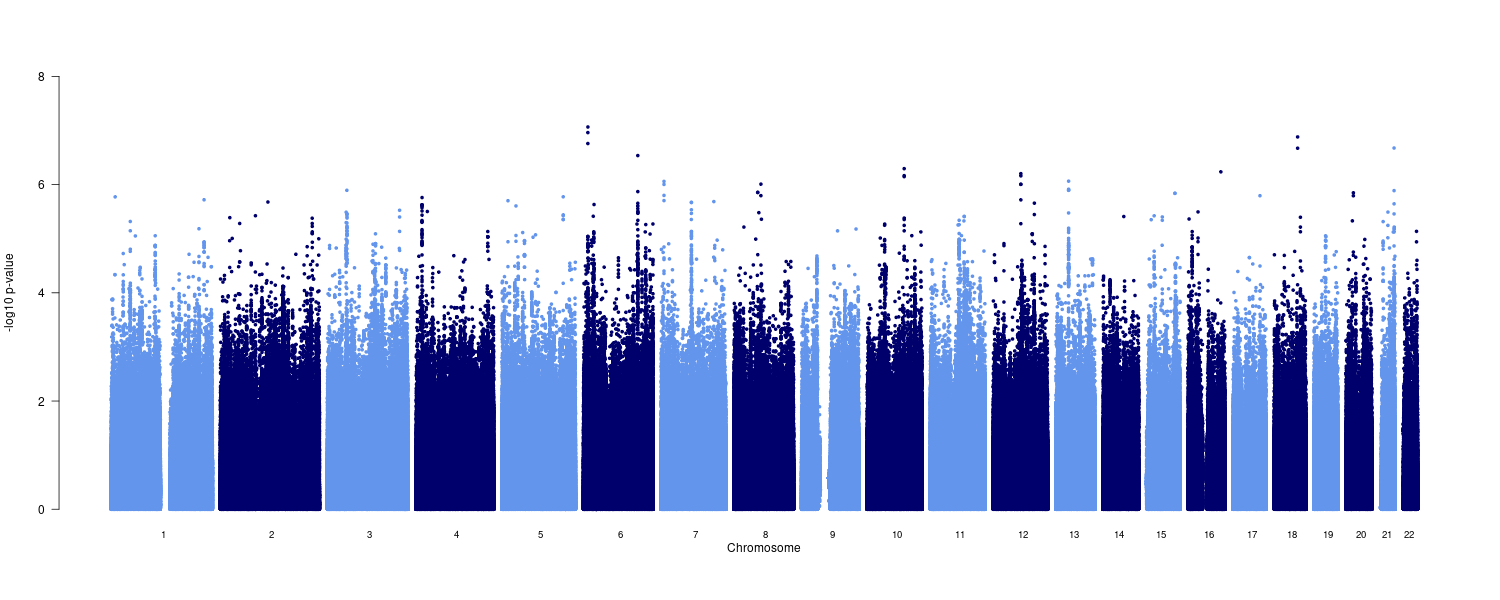


HOS vs NOT in META


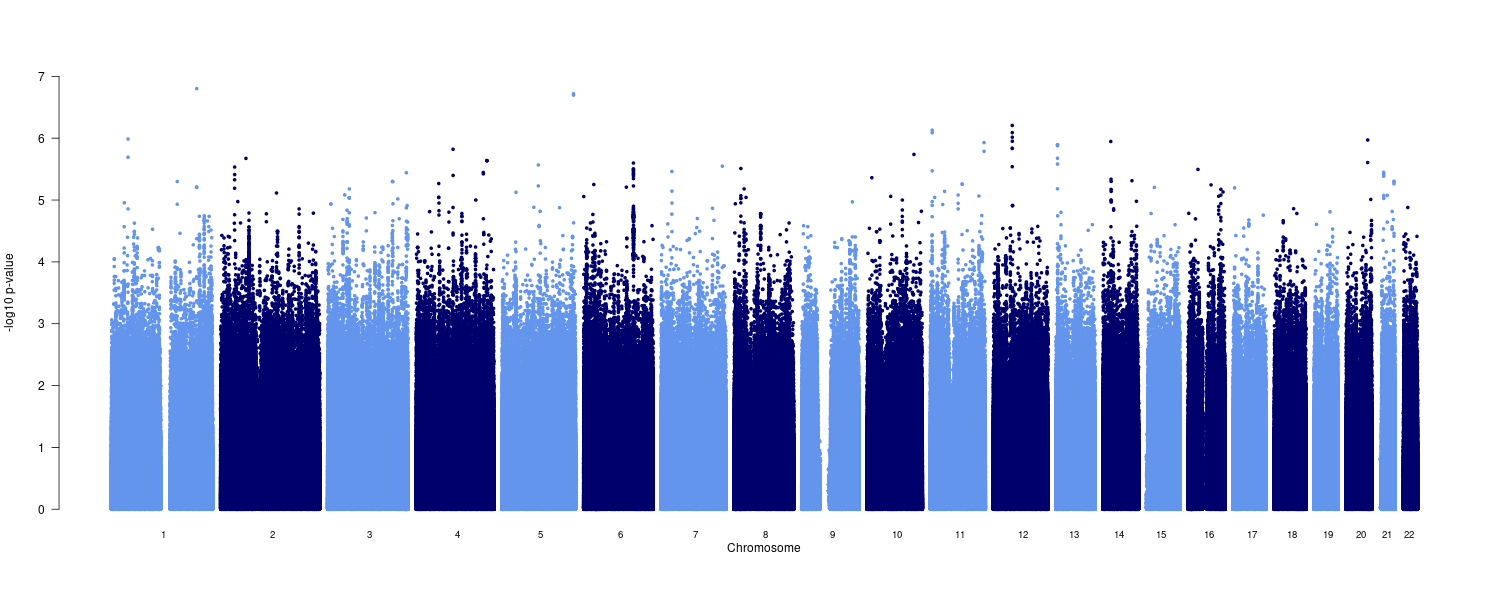


HOS vs NOT in WHITE


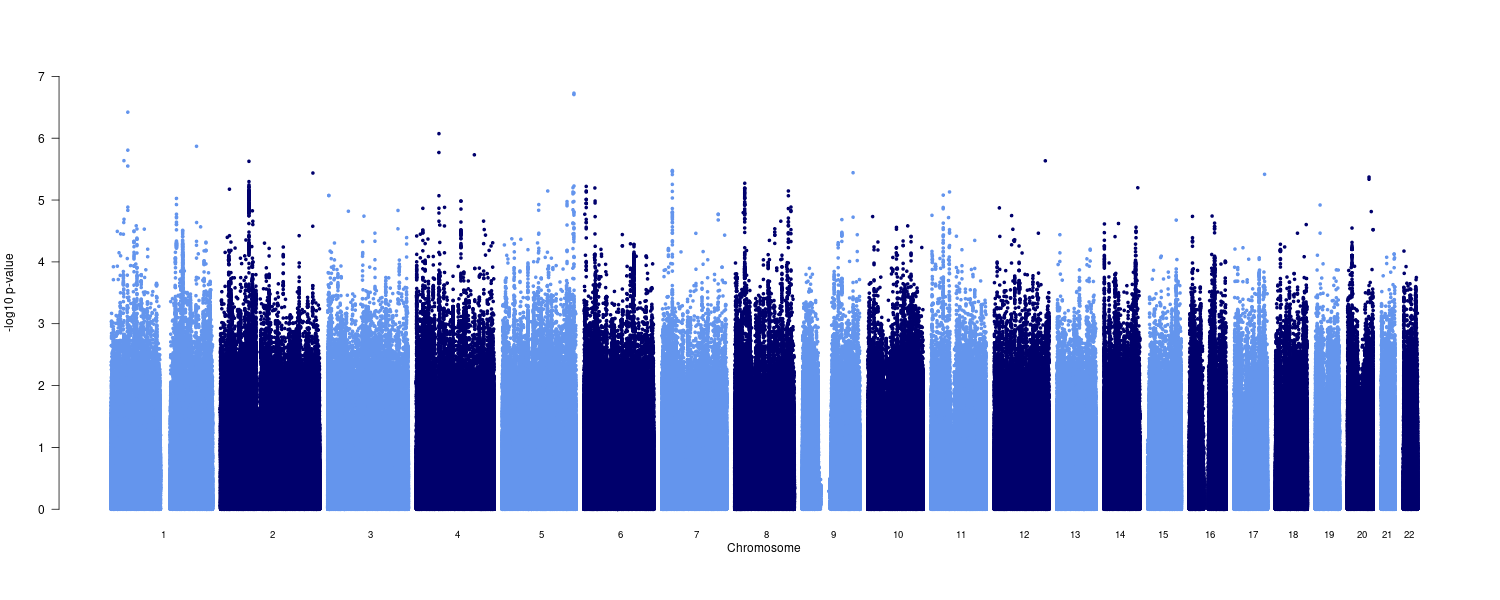


HOS vs NOT in BLACK


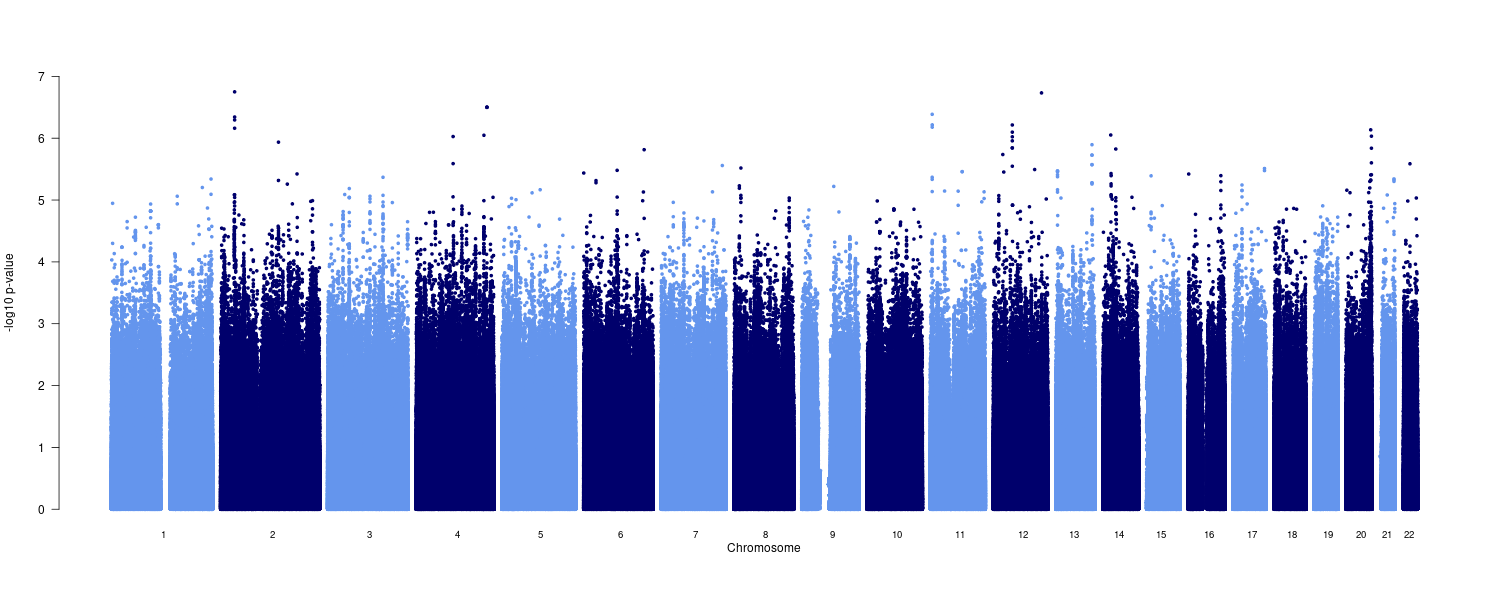


HOS vs NOT in HISPANIC


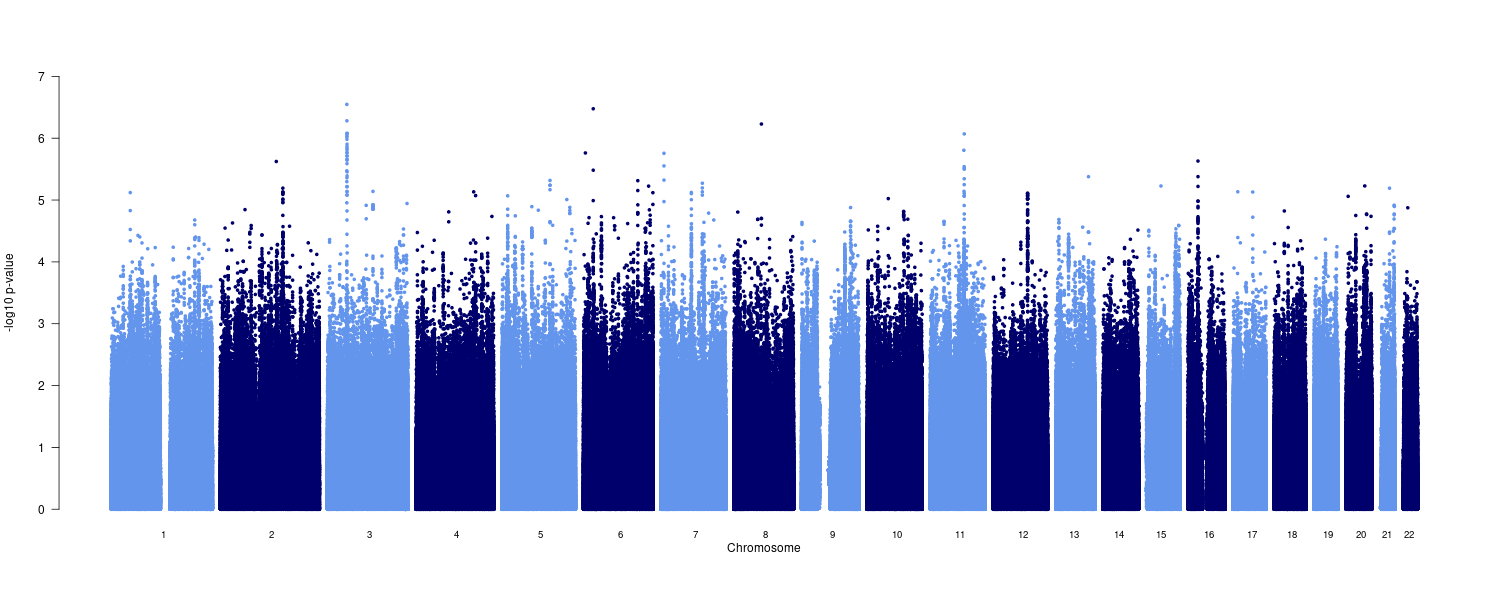


SEV vs POP in META


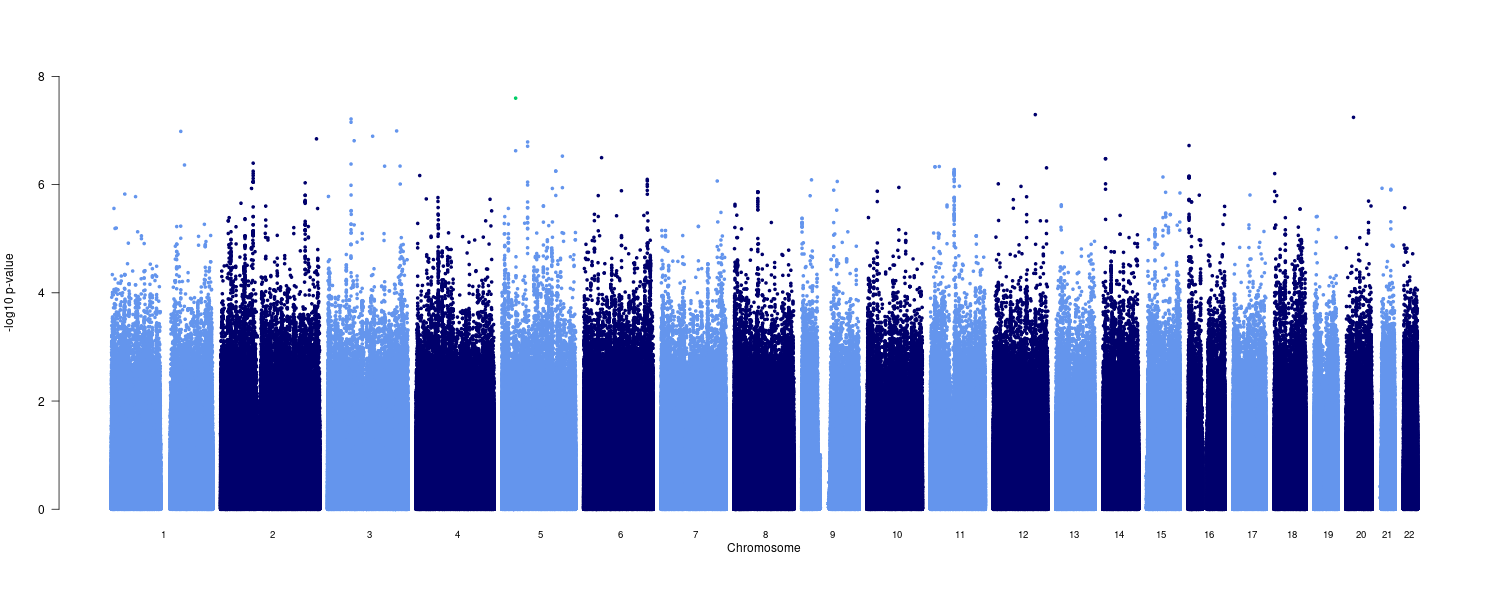


SEV vs POP in WHITE


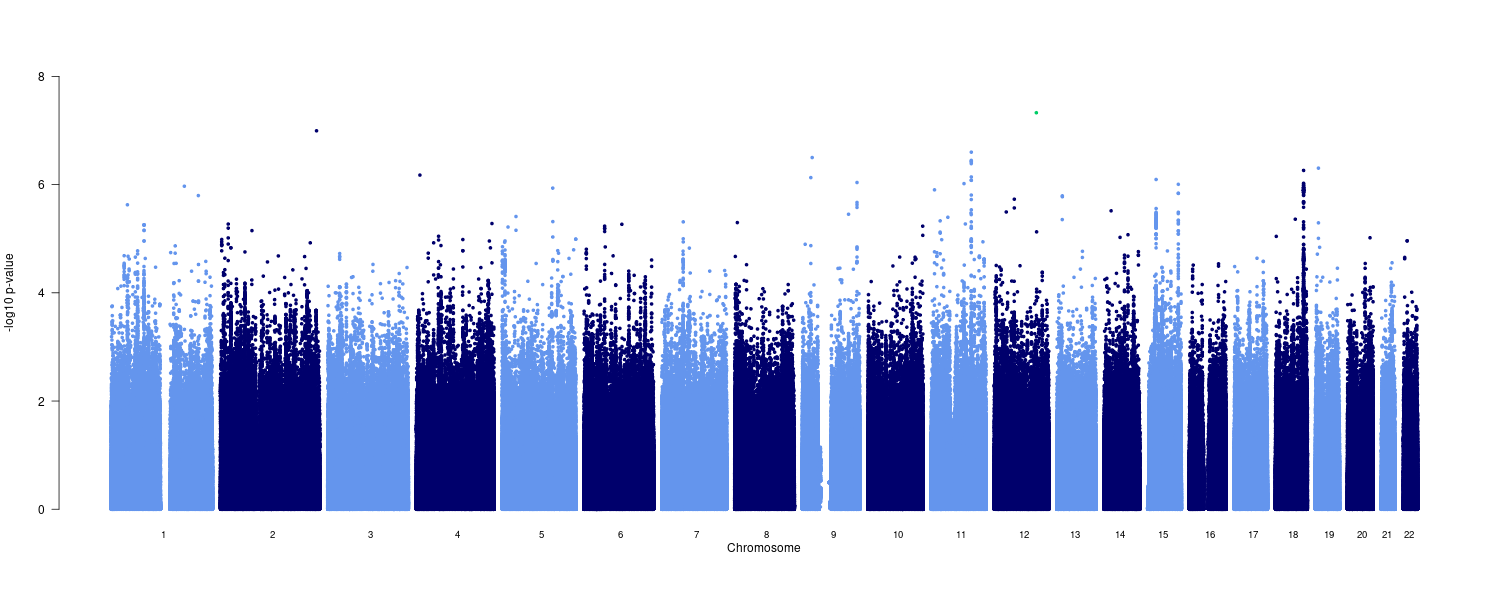


SEV vs POP in BLACK


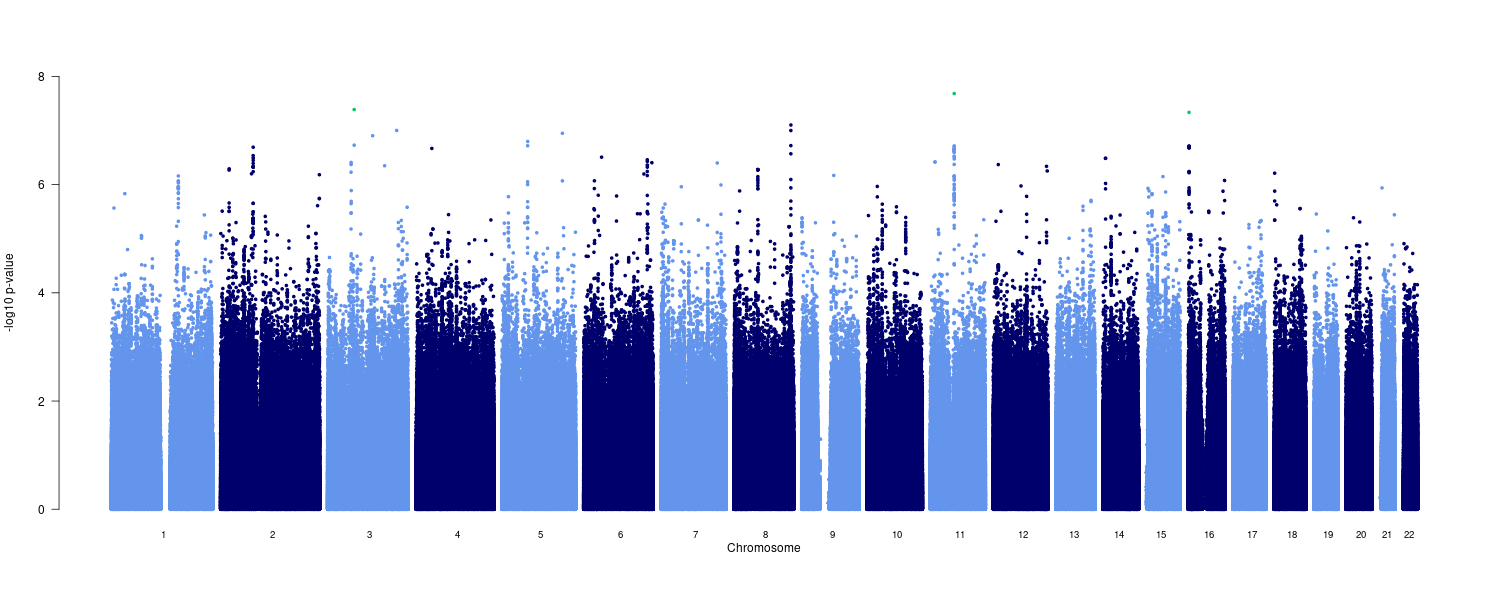


SEV vs POP in HISPANIC


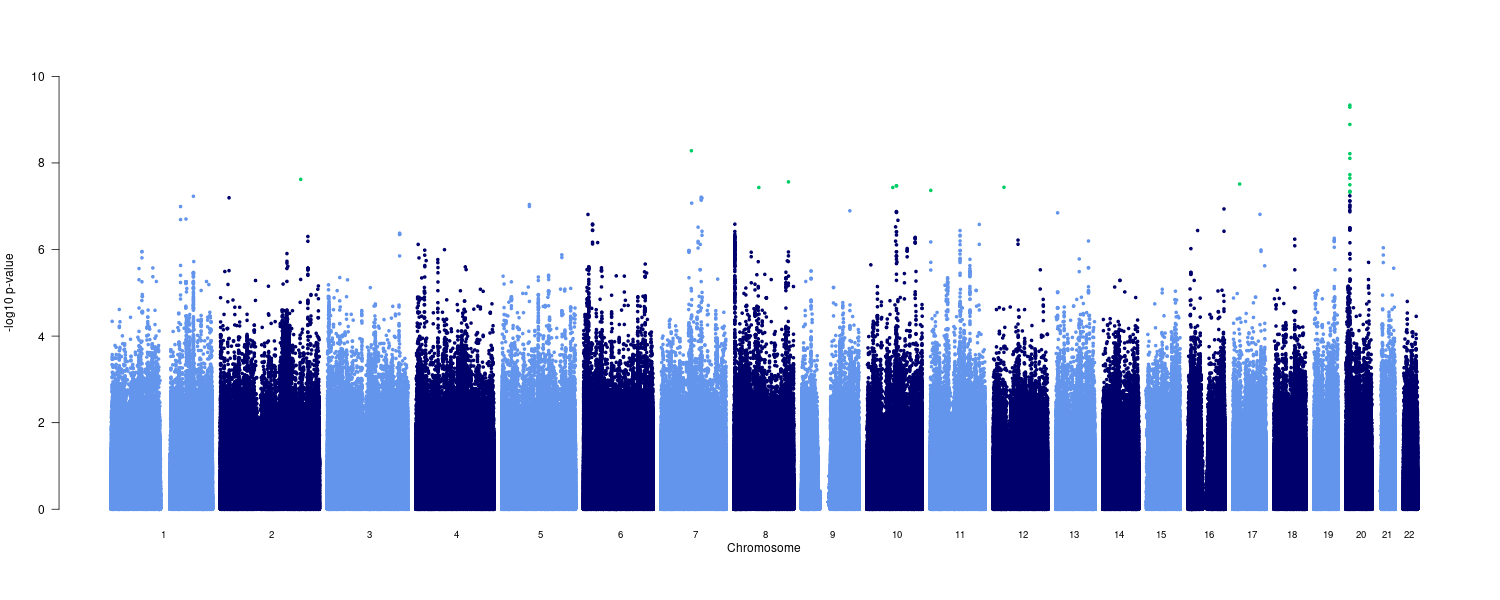


**Figure S3 – LocusZoom plots within each HARE group for COVID-19 Positivity (POS vs POP).**

*gray color indicates that LD information not available for this SNP in the reference population

rs73910904 in WHITE

rs73910904 in BLACK

rs73910904 in HISPANIC

rs73062389 in WHITE

rs73062389 in BLACK

rs73062389 in HISPANIC

rs60870724 in WHITE*

rs60870724 in BLACK

rs60870724 in HISPANIC

rs8176719 in WHITE*

rs8176719 in BLACK*

rs8176719 in HISPANIC*

**Figure S4 – Forest plots of significantly associated SNPs across outcomes**

**
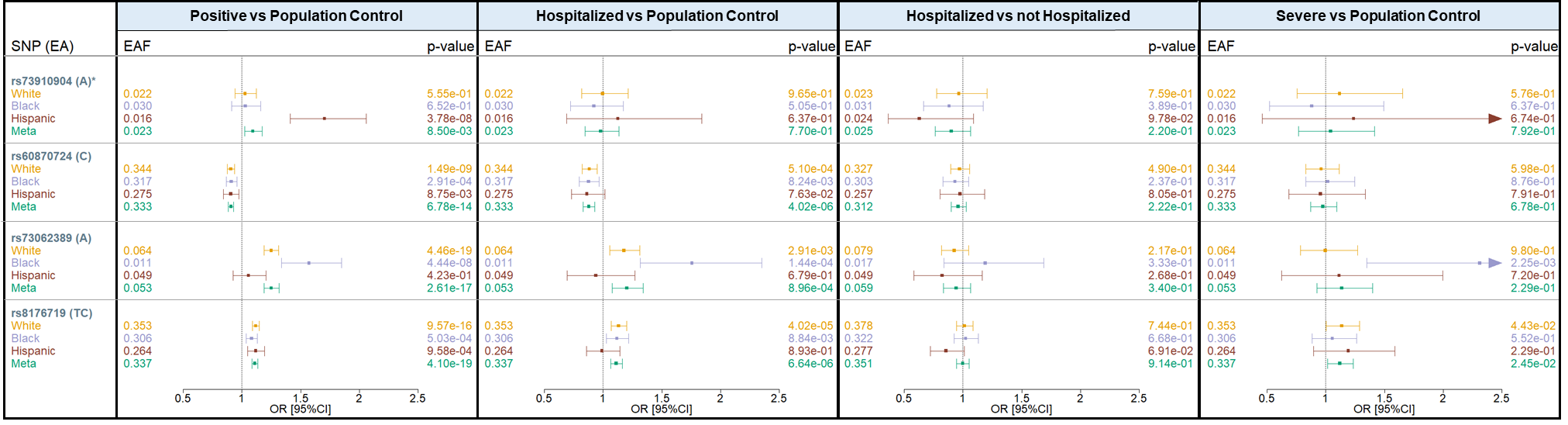
**

EA = effect allele, EAF = effect allele frequency, OR = odds ratio; Meta=Multi-population meta-analysis

**Figure S5. Multi-marker Analysis of GenoMic Annotation (MAGMA) analysis prioritizes multiple genes near rs60870724.**


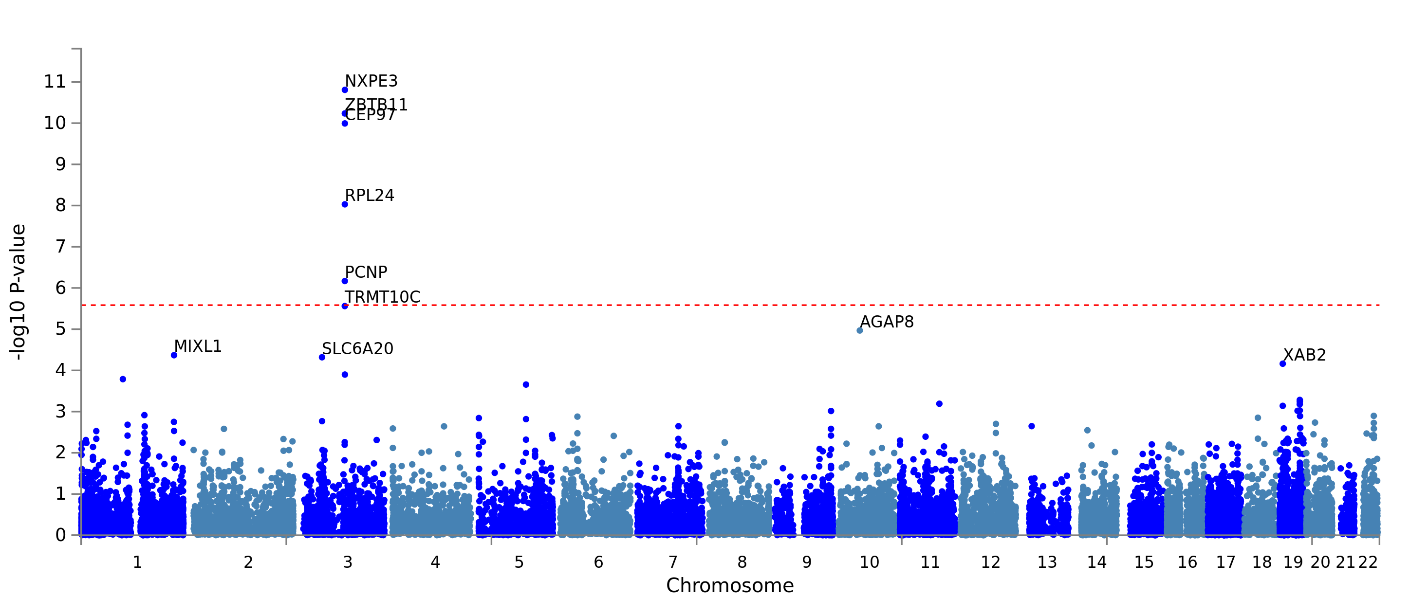


Red line represents Bonferroni significance level (0.05 / 19148 genes = 2.61 x 10^-6^). Top 10 gene associations were annotated, more details available in Table S4.

**Supplemental Tables**

**Table S1. Descriptive Statistics of Study Samples.**

Age is presented as mean ± SD, all other numbers are counts.

|  | **White** | **Black** | **Hispanic** |
| --- | --- | --- | --- |
| Age | 62.22±13.43 | 56.53±12.05 | 53.63±15.37 |
| Male | 338447 | 84462 | 4602 |
| COVID-19 Positive | 11778 | 4893 | 2497 |
| Death | 222 | 63 | 40 |
| Severe | 321 | 221 | 80 |
| Moderate | 1984 | 1036 | 41100 |
| Mild | 9251 | 3573 | 1962 |
| Hospitalized | 2417 | 1300 | 517 |
| Severe COVID-19 | 543 | 284 | 120 |
| Population Controls |  |  |  |
| For Positivity | 357198 | 94556 | 41100 |
| For Hospitalized | 366449 | 98129 | 43062 |
| For Severe | 368433 | 99165 | 43477 |

**Table S2. Genome-wide significant results for COVID-19 positivity in each HARE-assigned group.**

| **rsid**  **chr:pos** | **HARE group** | **EAF** | **OR** | **95% CI** | **p-value** | **Imputation R^2^** | **het**  **p-value** | **I^2^** |
| --- | --- | --- | --- | --- | --- | --- | --- | --- |
| rs73910904*  2:6153442:G:A | Meta | 0.023 | 1.094 | (1.02, 1.17) | 0.009 |  | 6.51E-06 | 0.92 |
|  | White | 0.022 | 1.027 | (0.94, 1.12) | 0.555 | 0.960 |  |  |
|  | Black | 0.030 | 1.028 | (0.91, 1.16) | 0.652 | 0.949 |  |  |
|  | Hispanic | 0.016 | 1.705 | (1.41 , 2.06) | 3.78E-08 | 0.966 |  |  |
| rs73062389  3:45835417:G:A | Meta | 0.053 | 1.249 | (1.19 , 1.31) | 2.61E-17 |  | 0.0056 | 0.81 |
|  | White | 0.064 | 1.249 | (1.19 , 1.31) | 4.46E-19 | 0.991 |  |  |
|  | Black | 0.011 | 1.571 | (1.34 , 1.85) | 4.44E-08 | 0.995 |  |  |
|  | Hispanic | 0.049 | 1.056 | (0.92 , 1.21) | 4.23E-01 | 0.999 |  |  |
| rs60870724  3:101495330:CA:C | Meta | 0.333 | 0.906 | (0.88 , 0.93) | 6.78E-14 |  | 0.9841 | 0 |
|  | White | 0.344 | 0.905 | (0.88 , 0.93) | 1.49E-09 | 0.734 |  |  |
|  | Black | 0.317 | 0.91 | (0.86 , 0.96) | 2.91E-04 | 0.750 |  |  |
|  | Hispanic | 0.276 | 0.904 | (0.84 , 0.97) | 8.75E-03 | 0.775 |  |  |
| rs8176719  9:136132908:T:TC | Meta | 0.337 | 1.109 | (1.08 , 1.13) | 4.10E-19 |  | 0.4948 | 0 |
|  | White | 0.353 | 1.118 | (1.09 , 1.15) | 9.57E-16 | 0.982 |  |  |
|  | Black | 0.306 | 1.082 | (1.03 , 1.13) | 5.03E-04 | 0.969 |  |  |
|  | Hispanic | 0.264 | 1.117 | (1.05 , 1.19) | 9.58E-04 | 0.980 |  |  |

chr:pos = chromosome and position hg19/GRCh37, EAF=effect allele frequency, OR = odds ratio, Meta=Multi-population meta-analysis, het p-value = heterozygosity p-value

* indicates that the SNP association was novel.

**Table S3 - Results of significant MVP SNPs in COVID-19 HGI Results (Release 5, leave out MVP and 23&Me)**

| **rsid** | **EA/ NEA** | **Analysis (HGI analysis number)** | **Population** | **EAF** | **beta** | **se** | **p-value** | **het_p** | **N studies** | **N samples** |
| --- | --- | --- | --- | --- | --- | --- | --- | --- | --- | --- |
| rs73910904 | A/G | Very severe respiratory confirmed covid vs. population (A2) | Meta | 0.021 | -0.011 | 0.085 | 8.94E-01 | 0.46 | 16 | 711,838 |
|  |  |  | White | 0.021 | -0.068 | 0.092 | 4.62E-01 | 0.53 | 14 | 707,407 |
|  |  | Hospitalized covid vs. not hospitalized covid (B1) | Meta | 0.080 | -0.104 | 0.104 | 3.20E-01 | 0.98 | 17 | 18,031 |
|  |  |  | White | 0.086 | -0.043 | 0.104 | 6.77E-01 | 0.95 | 12 | 16,645 |
|  |  | Hospitalized covid vs. population (B2) | Meta | 0.023 | -0.003 | 0.058 | 9.53E-01 | 0.81 | 28 | 1,286,529 |
|  |  |  | White | 0.024 | -0.004 | 0.062 | 9.50E-01 | 0.61 | 21 | 1,206,629 |
|  |  | Covid vs. population (C2) | Meta | 0.027 | 0.029 | 0.031 | 3.45E-01 | 0.95 | 44 | 1,668,694 |
|  |  |  | White | 0.028 | 0.006 | 0.032 | 8.41E-01 | 0.93 | 34 | 1,588,425 |
| rs73062389 | A/G | Very severe respiratory confirmed covid vs. population (A2) | Meta | 0.049 | 0.207 | 0.064 | 1.27E-03 | 0.48 | 14 | 701,379 |
|  |  |  | White | 0.049 | 0.174 | 0.069 | 1.17E-02 | 0.45 | 12 | 696,948 |
|  |  | Hospitalized covid vs. not hospitalized covid (B1) | White | 0.143 | -0.083 | 0.063 | 1.89E-01 | 0.94 | 8 | 13,562 |
|  |  | Hospitalized covid vs. population (B2) | White | 0.047 | 0.159 | 0.043 | 2.47E-04 | 0.61 | 16 | 903,334 |
|  |  | Covid vs. population (C2) | Meta | 0.055 | 0.214 | 0.023 | 3.49E-21 | 0.40 | 38 | 1,313,728 |
|  |  |  | White | 0.056 | 0.220 | 0.022 | 8.28E-23 | 0.72 | 30 | 1,272,287 |
| rs8176719 | TC/T | Very severe respiratory confirmed covid vs. population (A2) | Meta | 0.386 | 0.122 | 0.031 | 7.99E-05 | 0.03 | 15 | 703,239 |
|  |  |  | White | 0.387 | 0.145 | 0.035 | 2.87E-05 | 0.02 | 12 | 696,948 |
|  |  | Hospitalized covid vs. not hospitalized covid (B1) | Meta | 0.359 | -0.022 | 0.031 | 4.77E-01 | 0.62 | 14 | 16,974 |
|  |  | Hospitalized covid vs. population (B2) | Meta | 0.373 | 0.094 | 0.019 | 9.39E-07 | 0.02 | 24 | 1,266,057 |
|  |  |  | White | 0.372 | 0.116 | 0.022 | 1.41E-07 | 0.003 | 15 | 1,174,804 |
|  |  | Covid vs. population (C2) | Meta | 0.370 | 0.095 | 0.010 | 1.92E-21 | 0.10 | 40 | 1,536,911 |
|  |  |  | White | 0.369 | 0.105 | 0.011 | 1.70E-22 | 0.06 | 28 | 1,445,208 |

EA=effect allele, NEA=non-effect allele, EAF=effect allele frequency, beta= beta of logistic regression, se=standard error of beta, het_p = heterozygosity p-value, Meta=multi-population meta-analysis. * rs6087072 was not available in the HGI dataset

**Table S4. Top 10 genes for each outcome from Multi-marker Analysis of GenoMic Annotation (MAGMA) analysis.**

See Excel Document

**Table S5. Top 10 gene sets for each outcome.**

See Excel Document

**Table S6. Previously reported SNPs with COVID-19 phenotypes in the Million Veteran Program (MVP).**

See Excel Document

**Table S7. Comparison between ABO genotype calling and ABO clinical labs test**

| **ABO_geno** | **ABO_labs** | **N** | **%** |
| --- | --- | --- | --- |
| **Informative clinical blood type** | | |  |
| O | O | 249 | 46.80% |
| A | A | 169 | 31.77% |
| B | B | 79 | 14.85% |
| AB | AB | 25 | 4.70% |
| AB | B | 1 | 0.19% |
| A | O | 2 | 0.38% |
| AB | A | 1 | 0.19% |
| **Non-Informative blood type** | |  |  |
| A | NP | 2 | 0.38% |
| A | POS | 1 | 0.19% |
| AB | AB/D | 1 | 0.19% |
| B | NP | 1 | 0.19% |
| O | POS | 1 | 0.19% |
| **Total** |  | **532** |  |

**Table S8. Association of ABO blood type with COVID-19 outcomes.**

See Excel Document
